# Hsp90 middle domain phosphorylation initiates a complex conformational program to recruit the ATPase-stimulating cochaperone Aha1

**DOI:** 10.1038/s41467-019-10463-y

**Published:** 2019-06-12

**Authors:** Wanping Xu, Kristin Beebe, Juan D. Chavez, Marta Boysen, YinYing Lu, Abbey D. Zuehlke, Dimitra Keramisanou, Jane B. Trepel, Christosomos Prodromou, Matthias P. Mayer, James E. Bruce, Ioannis Gelis, Len Neckers

**Affiliations:** 1 Urologic Oncology Branch, National Cancer Institute, Bethesda, MD 20892 USA; 2 Department of Genome Sciences, University of Washington School of Medicine, Seattle, WA 98195 USA; 3 Center for Molecular Biology, University of Heidelberg, 69120 Heidelberg, Germany; 4 Department of Chemistry, University of South Florida, Tampa, FL 33620 USA; 5 Developmental Therapeutics Branch, National Cancer Institute, Bethesda, MD 20892 USA; 6 Genome and Damage Stability Center, University of Sussex, Brighton, BN1 9RH UK; 7 Present Address: Center for Therapeutic Research of Hepatocarcinoma, Beijing 302 Hospital, 100 Xi Si Huan Middle Road, 100039 Beijing, China

**Keywords:** Chaperones, Mass spectrometry, Phosphorylation, Solution-state NMR

## Abstract

Complex conformational dynamics are essential for function of the dimeric molecular chaperone heat shock protein 90 (Hsp90), including transient, ATP-biased N-domain dimerization that is necessary to attain ATPase competence. The intrinsic, but weak, ATP hydrolyzing activity of human Hsp90 is markedly enhanced by the co-chaperone Aha1. However, the cellular concentration of Aha1 is substoichiometric relative to Hsp90. Here we report that initial recruitment of this cochaperone to Hsp90 is markedly enhanced by phosphorylation of a highly conserved tyrosine (Y313 in Hsp90α) in the Hsp90 middle domain. Importantly, phosphomimetic mutation of Y313 promotes formation of a transient complex in which both N- and C-domains of Aha1 bind to distinct surfaces of the middle domains of opposing Hsp90 protomers prior to ATP-directed N-domain dimerization. Thus, Y313 represents a phosphorylation-sensitive conformational switch, engaged early after client loading, that affects both local and long-range conformational dynamics to facilitate initial recruitment of Aha1 to Hsp90.

## Abstract (web summary)

Phosphorylation of Tyr313 in Hsp90 enhances the binding to its activator Aha1, but the underlying mechanism is unknown. Here, the authors study the structural consequences of Tyr313 phosphorylation, showing that it serves as a conformational switch in Hsp90 that enables Aha1 recruitment.

## Introduction

The molecular chaperone heat shock protein 90 (Hsp90) is essential for cellular proteostasis in eukaryotes. It facilitates the maturation, stability, and activity of >200 Hsp90-dependent proteins, termed clients. The chaperone performs these functions by collaborating with an array of cellular proteins referred to as cochaperones^1^.

Hsp90 is composed of an ATP-binding N-terminal domain (Hsp90-N) that attains ATPase competence upon structural rearrangement of the protein, a middle domain (Hsp90-M) involved in client interaction, and a C-terminal domain (Hsp90-C) mediating homodimerization. Hsp90-N and Hsp90-M are joined by an unstructured region of variable length consisting of mainly charged amino acids and referred to as the charged linker^2^. All three Hsp90 domains interact with various cochaperones. Aha1 can interact simultaneously with both Hsp90-M and Hsp90-N^3–6^.

Aha1 is the primary activator of Hsp90 ATPase activity^7^, and is comprised of an N-terminal domain (Aha1-N) and a C-terminal domain (Aha1-C) which are connected by a flexible linker. Aha1-N interacts with Hsp90-M in a nucleotide-independent manner, while the interaction between Aha1-C and Hsp90-N is significantly enhanced by ATP binding to Hsp90-N, as is the interaction between full length Aha1 and Hsp90^3,4^. Binding of Aha1 facilitates conformational changes in Hsp90 necessary to establish transient N-domain dimerization, including rearrangement and re-orientation of the catalytic loop in Hsp90-M, repositioning of Hsp90 N-domain and M-domain, and facilitation of ATP lid closure in Hsp90-N, leading to marked stimulation of Hsp90 ATPase activity^6,8^.

Hsp90 is constitutively dimerized via the C-terminal domain of each protomer. In the nucleotide-free state Hsp90 adopts a V-shaped conformation in which the N-domains of each protomer are not associated. ATP binding biases the chaperone toward transient closure of the N-domains, resulting in a conformation that is competent for ATP hydrolysis. Post-hydrolysis, Hsp90 returns to an open conformation. It has been suggested that the dwell time in each state is crucial for Hsp90 to optimally process its client proteins^3,9^. As the major Hsp90 ATPase accelerator in the cell, Aha1 thus plays a key role in regulating the timing of the Hsp90 chaperone cycle.

Post-translational modifications (PTMs), including phosphorylation, acetylation, S-nitrosylation, oxidation, ubiquitination, and sumoylation, are increasingly appreciated as regulating distinct aspects of Hsp90 function in eukaryotic cells, particularly in metazoans^2,10–13^. Together with the appearance of cochaperones, an increasing variety and abundance of PTMs allows for precise tuning of the Hsp90 chaperone cycle in response to both diverse environmental cues and the needs of specific clients. Further, molecular modeling studies suggest that some Hsp90 PTMs serve as conformational switches that facilitate allosteric regulation of chaperone structure during the chaperone cycle^14^.

Adding to the complexity of Hsp90 regulation in higher eukaryotes, PTMs affecting particular cochaperones may synergize with PTMs of Hsp90. Thus, tyrosine phosphorylation of Cdc37, a cochaperone that interacts with Hsp90 client kinases, results in partial unfolding of its C-terminal domain, which in turn facilitates Hsp90 phosphorylation in the Hsp90 N-domain that regulates interaction with Cdc37^15^. Such synergistic interplay between Cdc37 and Hsp90 provides directionality to the Hsp90 cycle^16^.

We previously reported that Hsp90/Aha1 interaction is dramatically enhanced by phosphorylation of the highly conserved Y313 residue in Hsp90-M (amino acid numbering based on human Hsp90α)^16^. In that study, we used isothermal titration calorimetry (ITC) to show that phosphomimetic substitution (Y313E) increases Hsp90 affinity for Aha1 by ~3.5-fold. Consistent with this observation, Hsp90 complexed with Aha1 in vivo is greater than seven times more likely to be phosphorylated on Y313 compared to the general pool of the chaperone, suggesting a correlative relationship between Y313 phosphorylation and Aha1 binding in cells^16^. The current study was undertaken to explore the mechanistic basis underlying these observations.

Using several orthogonal techniques, we show that Hsp90-Y313 is a phosphorylation-sensitive conformational switch in the M-domain that acts early in the chaperone’s conformational cycle (after client loading) to stimulate Aha1 recruitment. Unexpectedly, this is accomplished by markedly enhancing Aha1-C affinity for Hsp90-M while the chaperone is still in an open conformation. This requires that Aha1-N and Aha1-C domains simultaneously bind to distinct surfaces on the M-domain of each Hsp90 protomer. As might be predicted from this model, Y313 modification is asymmetric and need not occur simultaneously on both Hsp90 protomers.

## Results

### Hsp90-Y313 phosphorylation promotes Aha1 C-domain binding

**Fig. 1:**
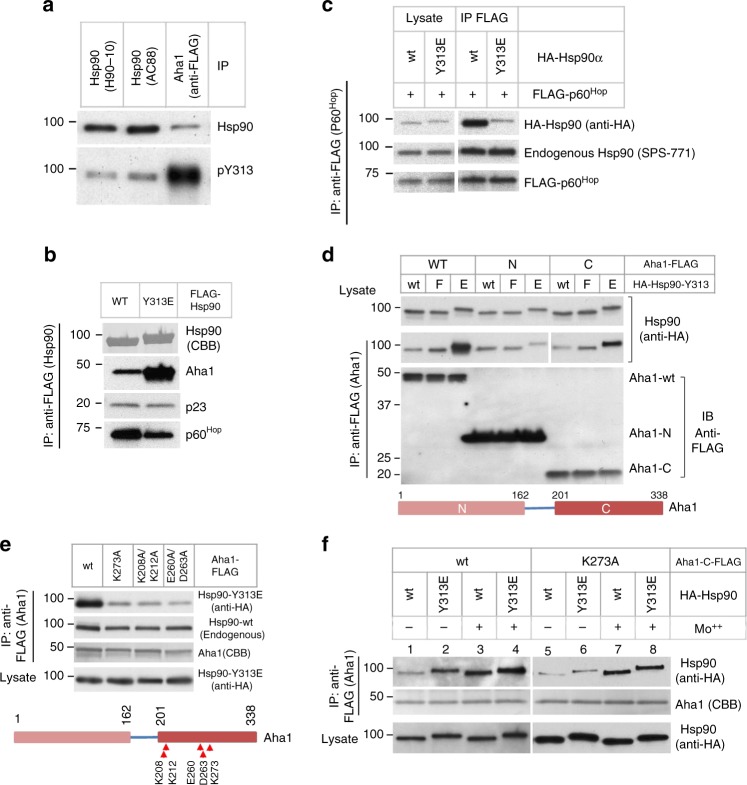
Y313 phosphorylation-promoted Hsp90–Aha1 interaction is mediated by Aha1-C. **a** Hsp90-associated Aha1 is phosphorylated on Y313 to a higher level compared to the general Hsp90 pool. Hsp90 was immunoprecipitated from transfected 293A cells with specific antibodies (H90-10 or AC88) or co-precipitated with FLAG-tagged Aha1. Hsp90 protein levels were examined by western blot, and Y313 phosphorylation was detected with an antibody specific for the phosphorylation of this site. **b** Y313 phosphomimetic substitution increases Hsp90 interaction with Aha1 but decreases the interaction with p60^Hop^. FLAG-tagged Hsp90 was immunoprecipitated from transfected cells. Co-precipitated endogenous Aha1, p23, and p60^Hop^ were detected by western blot. Precipitated Hsp90 proteins were stained with Coomassie Blue (CBB). **c** Y313E substitution decreases Hsp90 association with p60^Hop^. FLAG-p60^Hop^ was co-expressed with HA-Hsp90 and was immunoprecipitated from cells. Hsp90 and p60^Hop^ were detected by western blot with indicated antibodies. **d** Y313E phosphomimetic substitution in Hsp90 increases association of wild-type Aha1 and Aha1-C but not Aha1-N. FLAG-tagged Aha1 (wild-type or individual domains) were co-expressed with HA-tagged Hsp90, and complexes were immunoprecipitated with anti-FLAG resin. Exogenous Hsp90 was detected by western blot with anti-HA antibody. E = glutamic acid, F = phenylalanine. Bottom: schematic illustration of the Aha1 protein. **e** Point mutations in Aha1-C substantially decrease association with Hsp90-Y313E. Experiments were performed as in **d**. Endogenous co-precipitated Hsp90 (wild-type) was discerned with the antibody SPS-771, which significantly favors recognition of untagged Hsp90. Bottom: Schematic illustration of Aha1 with point-mutations indicated. **f** K273A mutation in Aha1-C abolishes the stimulatory effect of Y313 phosphomimetic substitution on Aha1 association. Experiments were performed as in **d**. Please see Supplementary Fig. 1 for more information. Source data for this figure are provided as a Source Data File

We showed previously that Hsp90 bound to Aha1 in cells was phosphorylated on Y313 to significantly higher levels than was the general intracellular pool of Hsp90 immunoprecipitated with the antibody AC88^16^. Since AC88 favors free Hsp90 that is not in complex with client proteins^17^, in the current study we tested Hsp90 precipitated using another antibody, H90-10, which recognizes Hsp90 in complex with clients^18^. We found that, like the Hsp90 immunoprecipitated with AC88, the Hsp90 fraction isolated by H90-10 immunoprecipitation was phosphorylated on Y313 to a substantially lesser degree than that seen for Hsp90 coprecipitated with Aha1 (Fig. 1a), confirming that Hsp90 in complex with Aha1 is preferentially phosphorylated on Y313 compared to the general cellular pool of the chaperone. Consistent with this observation, phosphomimetic substitution of Y313 with glutamic acid (Y313E) dramatically increased Hsp90 association with Aha1 (Fig. 1b).

Interestingly, while increasing Aha1 association, Y313E phosphomimetic substitution decreased Hsp90 association with another cochaperone, p60^Hop^ (Fig. 1b). This finding was confirmed by reciprocal immunoprecipitation, in which FLAG-p60^Hop^ and HA-Hsp90α were co-transfected into HEK293 cells. After FLAG immunoprecipitation followed by blotting for HA, we observed markedly less coprecipitating HA-Hsp90-Y313E compared to wild-type HA-Hsp90 (Fig. 1c). P60^Hop^ participates in the early, client loading step of the Hsp90 chaperone cycle (when Hsp90 N-domains are undimerized) and it competes with Aha1 for binding to Hsp90^2^. These data suggest that Y313E phosphomimetic substitution specifically affects an early transition step between dissociation of p60^Hop^ and recruitment of Aha1 to Hsp90.

In contrast to its impact on p60^Hop^ association with Hsp90, binding of the cochaperone p23 is minimally affected by Y313 phosphomimetic mutation (Fig. 1b). P23 binds most strongly to the N-domains of a fully closed (e.g., N-domain dimerized) Hsp90 dimer and thus stabilizes a late stage in the Hsp90 chaperone cycle at which point Hsp90 is capable of ATP hydrolysis^19,20^. However, ATP-dependent interaction of p23 with the middle domain of both yeast and human Hsp90 prior to N-domain dimerization has also been reported, although the functional consequences of this secondary interaction remain obscure^21–23^. Since it is technically difficult to visualize the population of human Hsp90 occupying the closed state (without cross-linking) even in the presence of ATP^24^, we considered the presence or absence of p23 in these Hsp90 immunoprecipitates to be uninformative for this study.

Human Aha1 comprises an N-terminal domain (Aha1-N, amino acid 1–162) and a C-terminal domain (Aha1-C, amino acid 201–338) connected by a linker of about 30 amino acids^3^. Each Aha1 domain folds well by itself and can independently interact with Hsp90^3,4,6^. To investigate which Aha1 domain is responsible for the increased interaction with Y313-phosphorylated Hsp90, we compared Aha1-N and Aha1-C association with wild-type and Y313-mutated Hsp90. Similar to results with full-length Aha1, a higher level of Hsp90 was detected in Aha1-C immunoprecipitates with the phosphomimetic Hsp90-Y313E compared to wild-type Hsp90 (Fig. 1d). However, this was not observed in Aha1-N immunoprecipitates. These results indicate that the enhanced interaction between Hsp90-Y313E and full length Aha1 is mediated by the C-terminal domain of Aha1. Importantly, these data are fully consistent with the Kd values of full-length Aha1, Aha1-N, and Aha1-C for both wild-type Hsp90 and Hsp90-Y313E (Supplementary Table 3). It is worth noting that a phenylalanine substitution of Y313 (Y313F) resulted in a mild increase in Hsp90 interaction with both full-length Aha1 and Aha1-C (Fig. 1d). This is likely due to a local change in Hsp90 conformation caused by disruption of a hydrogen bond between Y313 and D319 in Hsp90-M (see the section “Discussion”).

### The Aha1-C β-sheet mediates enhanced binding to Hsp90-Y313E

To further investigate the nature of Y313E-stimulated Hsp90–Aha1 interaction, we examined the structure of Aha1-C. NMR experiments have shown that the human Aha1-C domain in solution contains a β-sheet of five strands packed against a bundle of three α-helixes^4^ (Supplementary Fig. 1, PDB 1 X53[10.2210/pdb1X53/pdb]). Interestingly, the β-4 strand could be crosslinked to both Hsp90-N and Hsp90-M domains, and the lysine residue K273 in β-3 was protected by Hsp90 from reacting with chemically active reagents^3^, indicating that the β-sheet of Aha1-C interacts with Hsp90. To examine the potential involvement of this β-sheet in Hsp90 interaction promoted by Y313 phosphomimetic mutation, we made point mutations in the Aha1-C β-sheet and compared the association of these (full-length) mutants with Hsp90-Y313E to that of wild-type Aha1. At comparable expression levels, Aha1-C β-sheet mutants K273A, K208A/K212A and E260A/D263A displayed markedly less interaction with Hsp90-Y313E than did wild-type Aha1 (Fig. 1e, top panel), confirming that the Aha1-C β-sheet plays a key role in the enhanced interaction of full-length Aha1 with Hsp90-Y313E. Endogenous Hsp90 interaction was slightly reduced by these Aha1-C β-sheet mutations (compare top two panels in Fig. 1e), possibly reflecting the small amount of Y313 phosphorylation occurring on endogenous Hsp90.

Hsp90–Aha1 interaction is enhanced by ATP and its non-hydrolyzable analog AMPPNP. Molybdate, a phosphate analog, stabilizes an ATP-like Hsp90 conformation by occupying the binding site of the γ-phosphate after ATP hydrolysis^25,26^. To query the ATP dependence of Y313E-stimulated Hsp90–Aha1 interaction in cells, we compared Hsp90–Aha1 association between wild-type Aha1 and Aha1-K273A, in the presence and absence of molybdate. Molybdate markedly increased Hsp90 interaction with wild-type Aha1 (Fig. 1f, lanes 1 and 3), as expected. In contrast, even in the absence of molybdate Hsp90 Y313E mutation dramatically increased wild-type Aha1 interaction compared to wild-type Hsp90 (Fig. 1f, lanes 1 and 2). Interestingly, addition of molybdate caused a further increase in Aha1 association with Hsp90-Y313E, suggesting an additive effect with ATP (Fig. 1f, lanes 2 and 4). In the case of Aha1-K273A, on the other hand, Y313E mutation of Hsp90 failed to impact association of the two proteins, either in the presence or absence of molybdate (Fig. 1f, lanes 5, 6 and 7, 8). Together, these data suggest that the phosphomimetic Y313E mutation promotes Hsp90–Aha1 association by a mechanism that may act cooperatively with ATP, consistent with the possibility that phosphorylation of this residue may facilitate Aha1 recruitment during an early transition step between open and closed Hsp90 conformations.

### Aha1/Hsp90-Y313E binding does not require N-domain closure

**Fig. 2:**
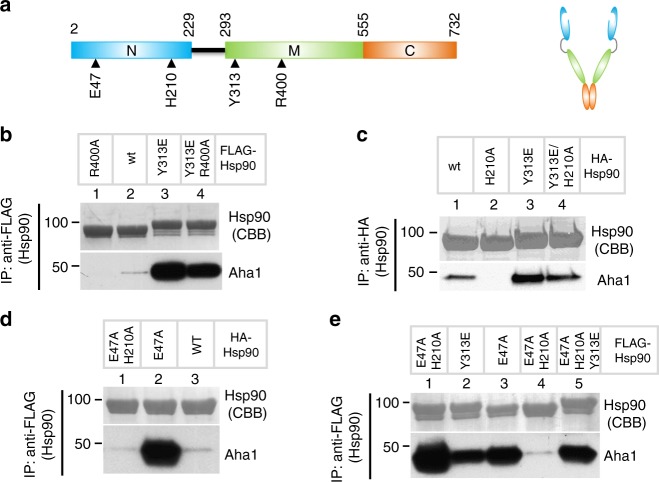
Aha1 interaction with Hsp90-Y313E is independent of Hsp90 N-domain closure. **a** Schematic illustration of Hsp90 monomer and dimer, showing the boundaries of the domains and the location of the mutations used in this figure. **b** Dimerization-inhibiting mutation in Hsp90 fails to counteract the stimulatory effect of Y313E on Aha1 interaction. FLAG-tagged Hsp90, wild-type, or indicated mutants were expressed in 293A cells, immunoprecipitated with the M2 anti-FLAG antibody and verified with CBB staining after electrophoresis. Co-precipitated Aha1 was examined by western blot. **c** The H210A mutation in Hsp90-N differentially affects Aha1 interaction with wild-type and Y313E Hsp90. Immunoprecipitation and western blot for indicated proteins were performed as in **b**. **d** The H210A mutation in Hsp90 completely abolishes the effect of the closed conformation-inducing Hsp90 mutation E47A on Aha1 association. Experiments were carried out as described in **b**. **e** Y313E and E47A in Hsp90 additively promote Aha1 interaction. The triple mutant E47A/H210A/Y313E displays partially restored Aha1 interaction. Experiments were performed as above. Please see Supplementary Fig. 2a, b for more information. Source data for this figure are provided as a Source Data File

The preceding data demonstrate that Y313E-induced Hsp90–Aha1 interaction is mediated by Aha1-C. Since Aha1-C is proposed to preferentially interact with the N-domain interface of an Hsp90 dimer in a closed (e.g., N-domain dimerized) conformation, we asked whether Y313 phosphomimetic mutation requires a closed conformation of Hsp90 to enhance Aha1 interaction. To evaluate this possibility, we made use of two Hsp90 point mutations (R400A, H210A) that interfere with N/M domain rearrangements required for N-domain dimerization (see Fig. 2a and Supplementary Table 1). The amino acid R380 in the M-domain of yeast Hsp90 contacts N-domain-bound ATP and facilitates closure of Hsp90 N-domains^27^. Likewise, mutation of the equivalent residue, R400, in human Hsp90 hinders formation of a stable, ATP-dependent rearrangement of Hsp90 N/M-domains that is required for ATP hydrolysis^27^. We found that R400A mutation abolished Aha1 interaction with wild-type Hsp90 (Fig. 2b, compare lanes 1 and 2). Significantly, inclusion of Y313E in an R400A Hsp90 mutant rescued Aha1 association (Fig. 2b, compare lanes 2–4). Together, these data suggest that Y313 phosphomimetic mutation promotes Aha1 interaction with N-domain undimerized Hsp90.

Specific binding between yeast Hsp90 N-domains and Aha1 C-domain has been observed in the presence of AMPPNP^4^. Using NMR, nucleotide-dependent changes in chemical shift in the presence of either Aha1-C or full-length Aha1 were detected at several locations within the Hsp90 N-domain, including helix 1, the ATP lid region, and at amino acid H197 (H210 in Hsp90α)^4^. H210 is located at the interface between the N-domain and M-domain of Hsp90, packing between E28, R32, R196, and F349 (Supplementary Fig. 2b), suggesting involvement of this amino acid in ATP-dependent N/M-domain rearrangement necessary for ATP hydrolysis. To test this possibility, we compared both the Aha1-binding affinity and the ATPase activity of wild-type Hsp90 and Hsp90-H210A. The Kd of Aha1 binding to Hsp90-H210A is increased by two-fold compared to wild-type Hsp90, while the Kd of AMPPNP binding is minimally affected by this mutation (Supplementary Table 2). As predicted, we found that H210A mutation reduced Hsp90-specific basal ATPase activity by >90% and, in contrast to wild-type Hsp90, this activity was not inducible by Aha1 (Supplementary Fig. 2a). These findings are consistent with the likelihood that Hsp90 N-domain and M-domain are unable to properly re-orient in the presence of H210A. Immunoprecipitation of FLAG-Hsp90 from HEK293 cells showed that H210A mutation abrogated Aha1 association with Hsp90. However, as was the case for mutation of R400, addition of an H210A mutation only modestly decreased Aha1 association with Hsp90-Y313E (Fig. 2c). Thus, two mutations that independently interfere with ATP-dependent N/M-domain rearrangement necessary for N-domain dimerization have minimal impact on enhanced Aha1 interaction with Hsp90 caused by phosphomimetic mutation of Y313, consistent with the hypothesis that Y313E facilitates an early step in Aha1 recruitment to N-domain undimerized Hsp90.

Hsp90 E47A (see Fig. 2a) is a mutation of the catalytic glutamic acid. Thus, this mutation does not interfere with ATP binding but does compromise ATP hydrolysis and stabilizes the N-domain dimerized, closed conformation of Hsp90^28^. Introduction of E47A dramatically increases Aha1 interaction with Hsp90 (Fig. 2d, compare lanes 2 and 3). Inclusion of H210A markedly reduced Aha1 interaction with Hsp90-E47A (Fig. 2d), in contrast to its minimal impact on Aha1 binding to Hsp90-Y313E (Fig. 2c, lanes 3 and 4). Similar to the additive effect of molybdate and Hsp90 Y313E mutation (Fig. 1f), Y313E and E47A mutations synergistically increase Aha1 association with Hsp90 (Fig. 2e, lanes 1–3). Importantly, inclusion of Y313E partially rescues Aha1 interaction with Hsp90-E47A/H210A (Fig. 2e, compare lanes 4 and 5). Together, these data suggest that Y313 phosphomimetic mutation induces an Hsp90 conformation that uniquely favors Aha1 interaction prior to N-domain dimerization, and this is mediated by enhanced binding of Aha1-C to Hsp90. This hypothesis is fully consistent with the Kd values, determined in the presence and absence of AMPPNP, of full-length Aha1, Aha1-N, and Aha1-C for both wild-type Hsp90 and Hsp90-Y313E (Supplementary Table 3). Intriguingly, these data show that the Kd of Aha1-C for Hsp90-Y313E is reduced by more than two-fold in the presence of AMPPNP, while the Kd of Aha1-C for binding to Hsp90-Y313E compared to wild-type Hsp90 (both in the presence of AMPPNP) is reduced by nearly seven-fold (Supplementary Table 3), consistent with an additive or cooperative role for ATP binding and Y313 phosphomimetic mutation (vide infra).

### Local and long-range conformational impact of Y313 mutation

**Fig. 3:**
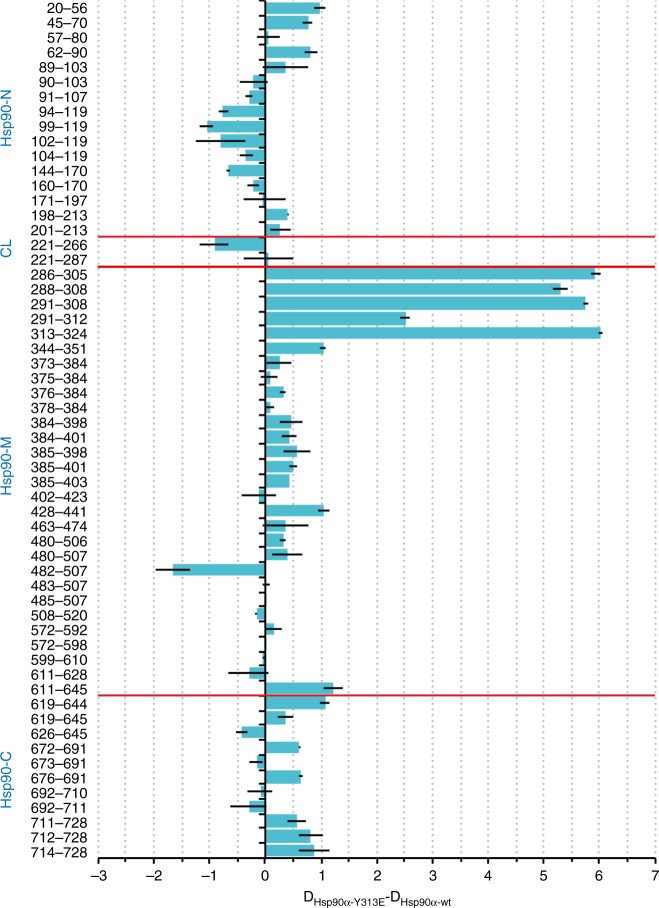
Y313 phosphomimetic substitution affects conformation of all Hsp90 domains. Hydrogen exchange mass spectrometry shows global impact of Y313E phosphomimetic substitution on Hsp90 conformation. Purified human Hsp90α (wild-type or Hsp90-Y313E) was diluted 1:20 into D_2_O buffer and incubated for 30 s at 30 °C in the absence of nucleotide. The reaction was quenched with ice cold quench buffer, the protein digested with immobilized pepsin, desalted and analyzed by liquid chromatography mass spectrometry (LC–MS). Deuteron incorporation into Hsp90-Y313E minus deuteron incorporation into wild-type Hsp90 is plotted for all peptic peptides (shown is the difference between each pair of means ± standard error for *n* = 3 independent experiments). For each peptide, bars depicting increased exchange for Hsp90-Y313E (compared to wild-type Hsp90) extend to the right, while bars showing decreased exchange for Hsp90-Y313E relative to wild-type Hsp90 extend to the left. Red lines indicate domain boundaries. CL, charged linker. The source data file contains the mean values ± SEM of deuteron incorporation for each corresponding peptide obtained from wild-type Hsp90 and Hsp90-Y313E, as well as the difference between the two sets of means ± SEM. The values underlying the means are no longer available

Taken together, these findings suggest Y313E mutation enhances Aha1 association by affecting Hsp90 conformation without promoting N-domain dimerization. To explore this possibility further, we employed hydrogen exchange-mass spectrometry (HDX) and NMR spectroscopy. The rate of exchange of amide protons yields information on solvent accessibility and hydrogen bonding of various parts of a protein in solution, enabling one to differentiate solvent exposed regions (higher exchange rate) from buried or highly structured regions (lower exchange rate)^29^, while signal broadening and chemical shifts of NMR spectra provide information on altered protein dynamic and structural properties. Collection and analysis of HDX data were performed in the absence of nucleotides. The most significant changes in hydrogen exchange and thus in conformational dynamics (altered solvent accessibility of Hsp90-Y313E compared to wild-type Hsp90) were observed in the region surrounding Y313 in the Hsp90-Y313E M-domain. Specifically, hydrogen exchange for Hsp90-Y313E in this region was dramatically increased, compared to wild-type Hsp90, particularly in peptides comprising aa286–308 (Fig. 3), which constitutes the extension from the C-terminal end of the charged linker and the turn leading to helix-11 in Hsp90-M, where Y313 resides (PDB 2CG9[10.2210/pdb2CG9/pdb], Supplementary Fig. 3a). Greater exchange was also observed for the peptide aa313–324, which includes the C-terminal half of helix-11 and the turn after it. In wild-type Hsp90, this loop–helix–loop module packs against a β-sheet comprised of five β-strands extending from amino acids 324 to 391 in Hsp90-M. It is very likely that this region becomes markedly more mobile in Hsp90-Y313E (perhaps contributing to the decreased migration of the protein, compared to wild-type Hsp90, in SDS gels). Indeed, the data suggest a moderate increase in solvent exposure of this β-sheet region, as evidenced by increased hydrogen exchange in peptides from aa344–351, 384–398, and 384–401.

**Fig. 4:**
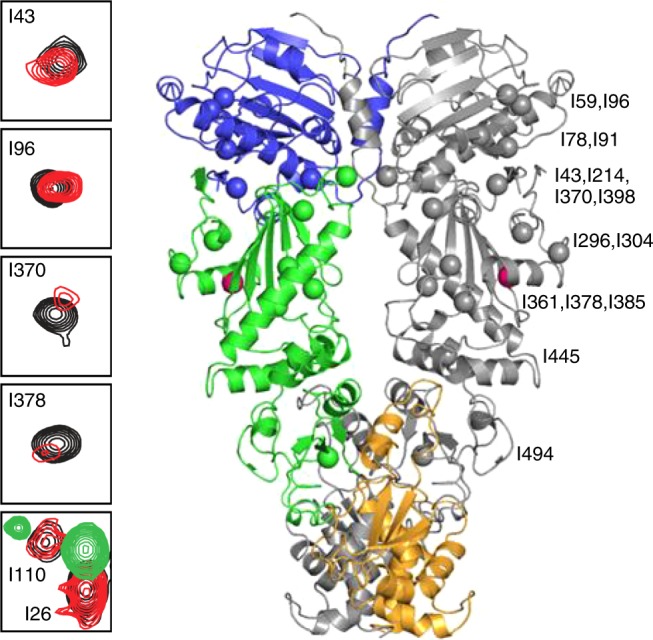
Y313E-induced conformational changes probed by methyl-TROSY NMR. Selected regions from the overlay of the ^1^H-^13^C-HMQC spectra of wild-type (black) and Hsp90-Y313E (red), showing signals that experience changes in chemical shifts and significant line broadening. The bottom panel includes an overlay of wild-type Hsp90 in the AMPPNP bound state (green) for I26 and I110 that undergo large chemical shift changes upon nucleotide addition due to Hsp90-N/N dimerization (see Supplementary Fig. 4). The Ile residues that experience significant chemical shift changes after phosphomimetic mutation mapped on the structure of Hsp90 in the closed state. One subunit is shown in gray, while in the other subunit N-, M- and C-domain of Hsp90 are colored blue, green, and orange, respectively. The site of the phosphomimetic mutation is shown in pink. Source data for this figure are provided as a Source Data File

NMR signal line widths were used to assess the impact of Y313 modification on the dynamics of Hsp90 that occur on a faster timescale than is measurable with HDX. Comparison of the ^13^C HMQC spectrum of isoleucine (Ile)-labeled Hsp90 to the spectrum of Hsp90-Y313E in the absence of nucleotide reveals that for a large number of Hsp90-M Ile residues that are not limited to the vicinity of Y313 (I296, I304, I361, I370, I378, I385, I398, I445, and I491), signals experience changes in chemical shift and severe line broadening (Fig. 4 and Supplementary Fig. 4a, b). Signal broadening is not a result of Hsp90-Y313 aggregation, as is evident by the comparison of its elution profile through an analytical gel-filtration column or the linewidths of signals from all three domains, with respect to wild-type Hsp90 (Supplementary Fig. 4c). Therefore, this phosphomimetic substitution has a profound impact on the structural and dynamic properties of Hsp90-M, causing a dramatic enhancement in millisecond timescale sampling of multiple conformations.

This altered Hsp90-M state affects the conformational properties of N-domain and C-domain, as well as N-/M-interdomain and M-/C-interdomain association, suggesting that Y313 phosphorylation elicits a global effect on the properties of Hsp90. In particular, the signals of I43, I214, and I398 which lie at the Hsp90-N/M interface show significant chemical shift changes (Fig. 4 and Supplementary Fig. 4a, b), indicating that Y313 phosphorylation induces a unique Hsp90-N/-M orientation relative to wild-type Hsp90. However, this reorientation of the Hsp90-N/M interface is not accompanied by inter-protomer dimerization of the N-domains in a manner that resembles the ATP-promoted closed conformation. Thus, the signals of I26, I33, and I110, which lie at the interface of dimerized N-domains and serve as excellent probes of the closed state, do not exhibit any change in chemical shift in Hsp90-Y313E relative to wild-type Hsp90 (Fig. 4 and Supplementary Fig. 4b).

In agreement with this observation, increased hydrogen exchange was detected in the region containing aa20–70, which comprises the C-terminal half of β-strand 1, helixes 1 and 2, and the turn in between the two helices (Fig. 3 and Supplementary Fig. 3a). Since β-strand 1 and helix 1 are less solvent-exposed in the closed (N-dimerized) conformation of the Hsp90 dimer (Supplementary Fig. 3a), increased hydrogen exchange in this region supports the hypothesis that Y313E phosphomimetic mutation hinders association of the two protomers. Increased exchange in helix 2, which is partially buried in the Hsp90 closed conformation, is consistent with this interpretation (Fig. 3 and Supplementary Fig. 3a). AMPPNP-induced association of Hsp90-Y313E N-domains further support this hypothesis. The NMR data show that, in the presence of AMPPNP, Hsp90-Y313E acquires a similar closed conformation as the one observed for wild-type Hsp90, as evidenced by the positions of I26, I33, I110 and other Hsp90-N signals (Supplementary Fig. 4d, e).

Compared to wild-type Hsp90, Hsp90-Y313E displayed decreased hydrogen exchange in the absence of nucleotide in the region of the ATP lid (peptide comprised of aa99–119, Fig. 3). Interestingly, decreased hydrogen exchange has been observed in the same region of human Hsp90β in the presence of both ATP and ADP^19^, suggesting the possibility of overlapping impact of nucleotide binding and Y313 phosphorylation on mobility of the Hsp90 ATP lid. Further, we observed chemical shift changes for Ile residues on the opposite side of the lid, specifically I59, I78, I91, and I96 (Fig. 4 and Supplementary Fig. 4a), suggesting that structural changes are propagated throughout the nucleotide-binding domain.

The notion of long-range conformational effects propagated by Y313 phosphomimetic substitution is also strengthened by altered hydrogen exchange observed in the Hsp90 C-terminal domain. Increased exchange in Hsp90-Y313E was detected for peptide aa611–645. However, a slight decrease was observed for the shorter peptide aa611–628 (Fig. 3), suggesting that the increase observed in the longer peptide fragment likely is due to aa629–645, a region that is buried in the fully closed conformation of wild-type Hsp90 (Supplementary Fig. 3a). Increased exchange was also observed in the C-terminal region (aa711–728) leading to the TPR domain-binding segment MEEVD (Fig. 3), consistent with the likelihood that the C-terminal tail of Hsp90-Y313E is more solvent exposed as a consequence of Y313 phosphorylation. Intriguingly, ATP binding to Hsp90-N results in similar deprotection of Hsp90β in this region^19^.

### Y313E mutation increases Hsp90-M/Aha1-C interaction

**Fig. 5:**
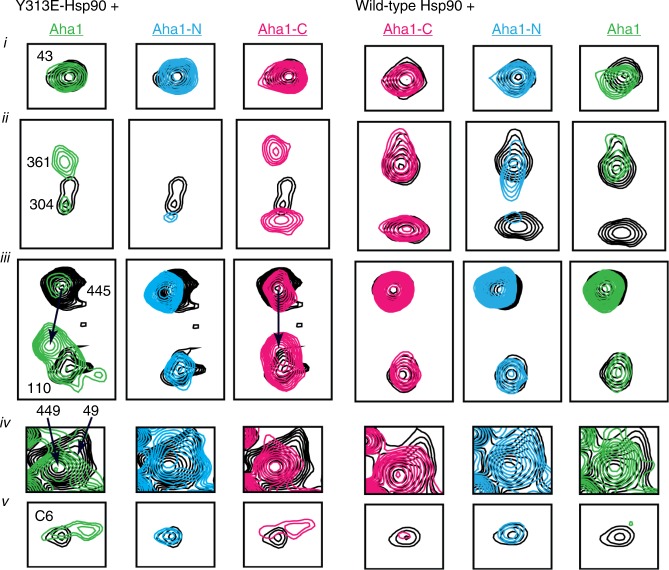
Modulation of Aha1–Hsp90 interaction by Y313E phosphomimetic substitution. Selected sets of signals from the titration of Y313E (left, black) or wild-type Hsp90 (right, black) with wild-type Aha1 (green), Aha1-N (cyan), or Aha1-C (pink). The lower panel shows the signal of an Ile residue from Hsp90-C for which no assignment is currently available (designated C6). Roman numbering denotes boxes extracted from the complete spectra shown in Supplementary Fig. 5

**Fig. 6:**
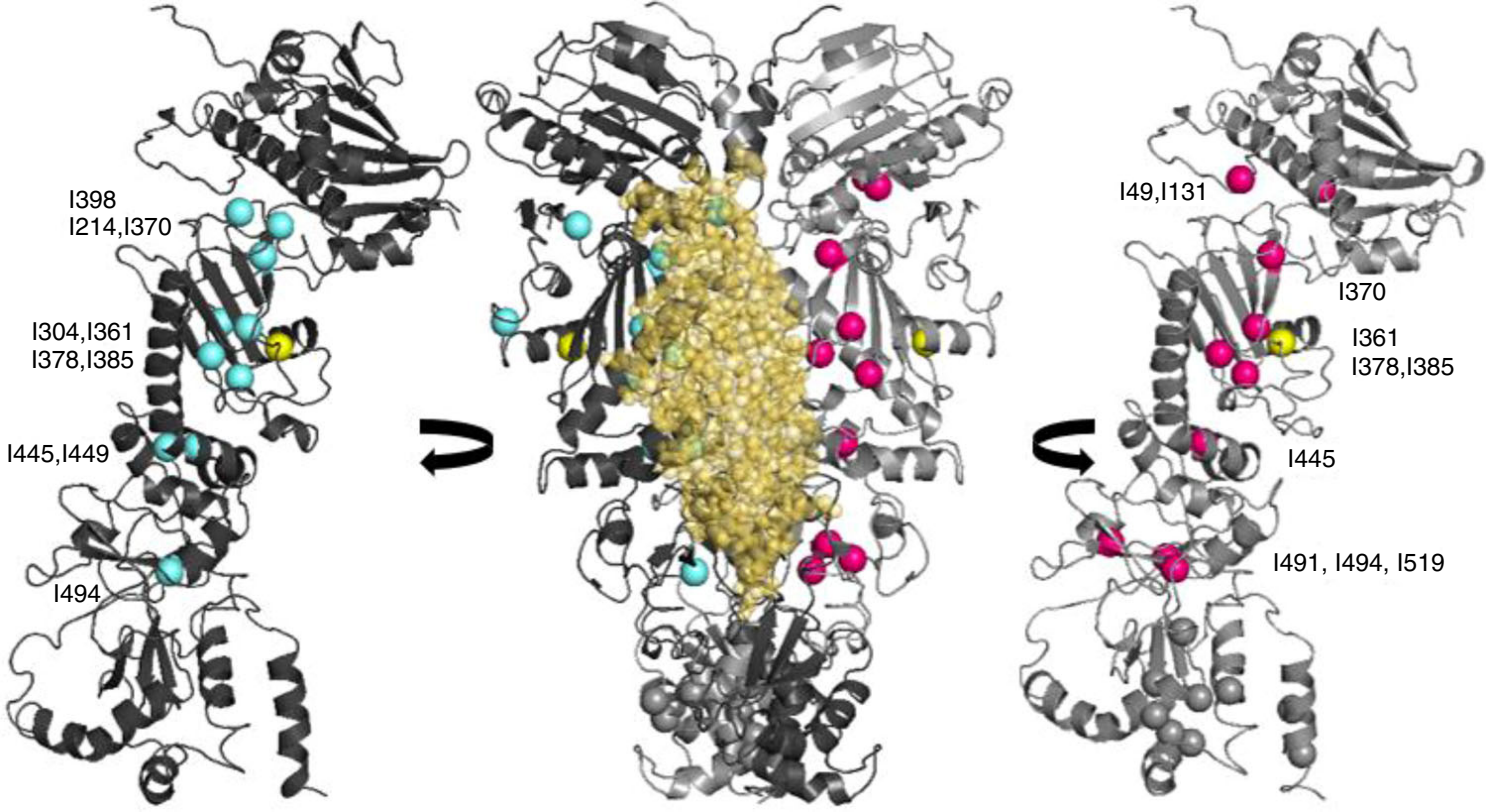
Aha1-N and Aha1-C chemical shift perturbations mapped on the Hsp90 closed dimer. Aha1-N affected Hsp90 residues are shown in cyan and Aha1-C affected residues are shown in pink. The closed Hsp90 dimer is shown in dark and light gray, and the position of the Y313 phosphomimetic mutation is shown in yellow. The position of Aha1-N is modeled in complex with one of the two protomers. All Hsp90-C Ile residues are depicted in gray

Aha1-C has been reported to interact with both N-domain and M-domain of Hsp90^3,4^. It has been proposed that interaction between Aha1-C and Hsp90-M is transient and Aha1-C switches to interact with Hsp90-N upon ATP binding and dimerization of the N-domains of each protomer^3^. Since phosphomimetic substitution of Y313 promotes Aha1 association via Aha1-C and does not appear to elicit or require the closed conformation of Hsp90, we hypothesized that this mutation may serve to facilitate Aha1-C interaction with Hsp90-M. To test this hypothesis, we monitored changes in the ^13^C HMQC spectrum of Ile-labeled Hsp90-Y313E in the presence of perdeuterated full-length Aha1, Aha1-N, or Aha1-C, and we compared the data obtained to the corresponding titrations of wild-type Hsp90. Indeed, addition of Aha1-C to Hsp90-Y313E causes prominent chemical shift changes to a large set of Ile signals from Hsp90-M (I361, I370, I378, I385, I445, I491, I494, I519), as well as several undefined Ile signals from Hsp90-C. Addition of Aha1-C to Hsp90-Y313E also causes small shift or broadening to I49 and I131 from Hsp90-N (Figs. 5, 6 and Supplementary Fig. 5). On the other hand, binding of Aha1-C has only a limited impact on signals obtained from wild-type Hsp90, including those of I361 and I445 from Hsp90-M, I43, I49, and I110 from Hsp90-N, as well as the same set of Hsp90-C signals that are perturbed when Hsp90-Y313E was titrated (Figs. 5, 6 and Supplementary Fig. 5).

Based on the forgoing data, we conclude that Aha1-C interacts in distinct ways with wild-type Hsp90 and the phosphomimetic mutant. Although Aha1-C binds in a dynamic fashion to multiple Hsp90 domains, the altered Hsp90-M conformation and relative domain orientation presented by Y313E-Hsp90 allows Aha1-C to interact preferentially with Hsp90-M. Titration with Aha1-N, however, does not reveal such a differential effect. As expected on the basis of the available structure of Hsp90-M in complex with Aha1-N, perturbations occur only for Ile signals of Hsp90-M and the N/M interface (I49, I214, I304, I361, I370, I378, I385, I398, I445, I449, and I494) (Figs. 5, 6 and Supplementary Fig. 5). With the exception of I304 and I361, which lie in the vicinity of the mutation, all other signals shift in the same direction for both wild-type and Hsp90-Y313E, suggesting that in contrast to Aha1-C, the mode of Aha1-N interaction with Hsp90 is not sensitive to, or slightly inhibited by, the presence of the phosphomimetic mutation. This interpretation of the data is fully consistent with the co-immunoprecipitation of individual Aha1 domains with wild-type Hsp90 and Hsp90-Y313E, as well as with the Kd values for full-length Aha1, Aha1-N and Aha1-C binding to either wild-type Hsp90 or Hsp90-Y313E, as described above.

When the titration of full-length Aha1 to Hsp90 is considered, the effect on the spectra of both wild-type and Hsp90-Y313E recapitulates a combined effect obtained from titration of the isolated domains, which is in agreement with the previously proposed globular nature of Aha1, where Aha1-N and Aha1-C domains are joined through a long, flexible linker. In this respect, signals that are uniquely affected by the addition of either Aha1-N or Aha1-C (e.g. I449 by Aha1-N and C6 by Aha1-C; C6 refers to an Ile residue in Hsp90-C for which no assignment is currently available) display similar changes in the presence of full-length Aha1 (Figs. 5, 6 and Supplementary Fig. 5). Despite the apparent overlap observed for the Aha1-N and Aha1-C binding sites on Hsp90-M, steric considerations suggest that initial binding of the two Aha1 domains more likely occurs via interaction with opposite faces of the M domains of the two Hsp90 protomers.

**Fig. 7:**
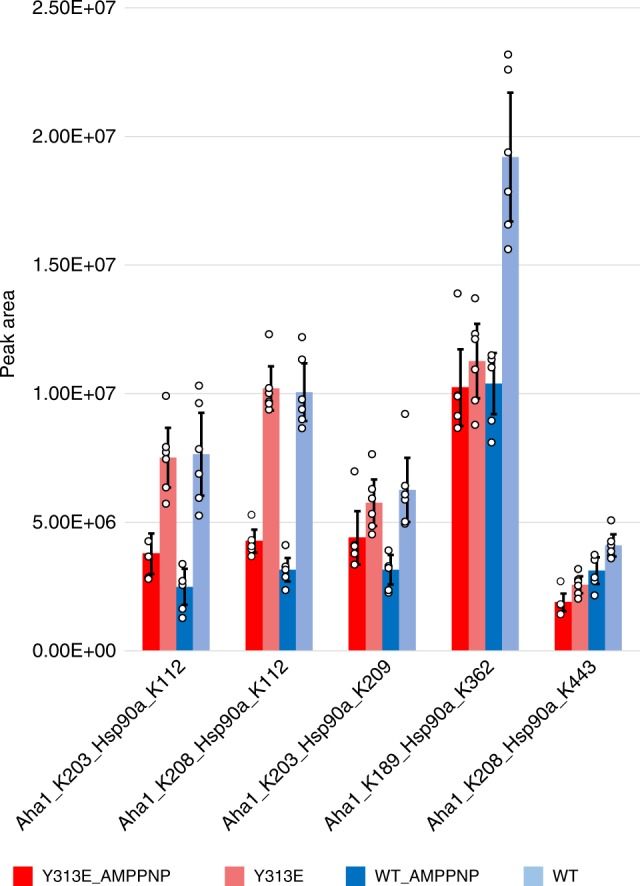
Hsp90–Y313E–Aha1 interaction profile shares similarities with Hsp90+ AMPPNP. Purified Hsp90α (wild-type or Y313E) and Aha1 proteins were mixed in the presence or absence of AMPPNP, followed by the addition of the crosslinking reagent BDP-NHP which crosslinks adjacent lysines (maximal cross-linking distance is 42.1 Å between the alpha-carbons of the linked lysine residues). Samples were then subjected to LC–MS analysis, crosslinked lysines were identified and the crosslink intensities were calculated and grouped for each crosslink pair. Error bars represent 95% confidence interval (*n* = 6 independent experiments). Please see Supplementary Fig. 3b, c for more information. Source data for this figure are provided as a Source Data File

To further explore the interaction between Aha1 and Hsp90, we examined lysine cross-linking between wild-type Hsp90, or Hsp90-Y313E, and Aha1, in the presence or absence of AMPPNP, using mass spectrometry. In total we identified 147 non-redundant cross-linked peptide pairs and selected 22 for targeted quantitative mass spectrometry using a parallel reaction monitoring (PRM) method (Supplementary Data 1). Five non-redundant cross-linked peptide pairs were quantified between Aha1 and Hsp90 (wild-type and Y313E) and reflected differences between wild-type Hsp90 and Hsp90-Y313E in response to AMPPNP (Fig. 7 and Supplementary Fig. 3b; note that the data in Supplementary Fig. 3b represent a ratio of values for either wild-type Hsp90 or Hsp90-Y313E obtained in the presence and absence of AMPPNP, while the individual integrated peak areas are shown in Fig. 7). Incubating Hsp90 with AMPPNP prior to Aha1 addition tended to decrease the relative levels of the Hsp90-Aha1 cross-links, agreeing with prior cross-linking observations for Aha1 and Hsp90β^3^. Generally, the AMPPNP-induced changes were greater for wild-type Hsp90 than for Hsp90-Y313E (Supplementary Fig. 3b). We found two cross-links between the C-terminal half of Aha1 and Hsp90-M (K189^Aha1^-K362^Hsp90^ and K208^Aha1^-K443^Hsp90^) for both Hsp90-Y313E and wild-type Hsp90 (Fig. 7 and Supplementary Fig. 3b). The K208^Aha1^-K443^Hsp90^ cross-link displayed a reduction in relative level after pretreatment of Hsp90 with AMPPNP, but this reduction was similar for both wild-type Hsp90 and Hsp90-Y313E (Supplementary Fig. 3b). In contrast, although AMPPNP reduced the relative level of cross-links between K189^Aha1^–K362^Hsp90^ (wild-type Hsp90), the relative level of cross-links between K189^Aha1^ and K362^Hsp90-Y313E^ in the absence of AMPPNP was very similar to that of wild-type Hsp90 in the presence of AMPPNP, and it changed little upon addition of this nucleotide to Hsp90-Y313E (Fig. 7). This can also be visualized by comparing the cross-link ratio data for this cross-link pair (Supplementary Fig. 3b). These data support the possibility that Hsp90-Y313E is poised for a more rapid transition (compared to wild-type Hsp90) from the open to the ATP-potentiated N-domain dimerized state.

The other three inter-protein cross-links that we found were between Aha1-C and Hsp90-N (K203^Aha1^-K112^Hsp90^, K208^Aha1^-K112^Hsp90^, and K203^Aha1^-K209^Hsp90^). Each of these three cross-links displayed a similar trend of reduced levels with AMPPNP, although at a larger magnitude for wild-type Hsp90 than for Hsp90-Y313E (Fig. 7 and Supplementary Fig. 3b). We also examined Hsp90 intra-protomer cross-links, comparing wild-type Hsp90 to Hsp90-Y313E, in the presence and absence of AMPPNP (Supplementary Fig. 3b, c). One intra-protomer cross-link between K58^Hsp90^-K112^Hsp90^, which spans the Hsp90 ATP-binding pocket, displayed a much larger change in the presence of AMPPNP for wild-type Hsp90 than for Hsp90-Y313E. Two additional intra-protomer cross-links, K362^Hsp90^-K292^Hsp90^ and K414^Hsp90^-K410^Hsp90^, showed substantial variations between wild-type Hsp90 and Hsp90-Y313E, as did several inter-protomer cross-links, K414^homodimer^, K443^homodimer^, and K631^homodimer^ (Supplementary Fig. 3b, c). Together, these results support a conformational impact of Y313 phosphomimetic mutation throughout all Hsp90 domains, some of which mimic those induced by AMPPNP.

### Hsp90-Y313E stimulation of Aha1 binding is asymmetric

**Fig. 8:**
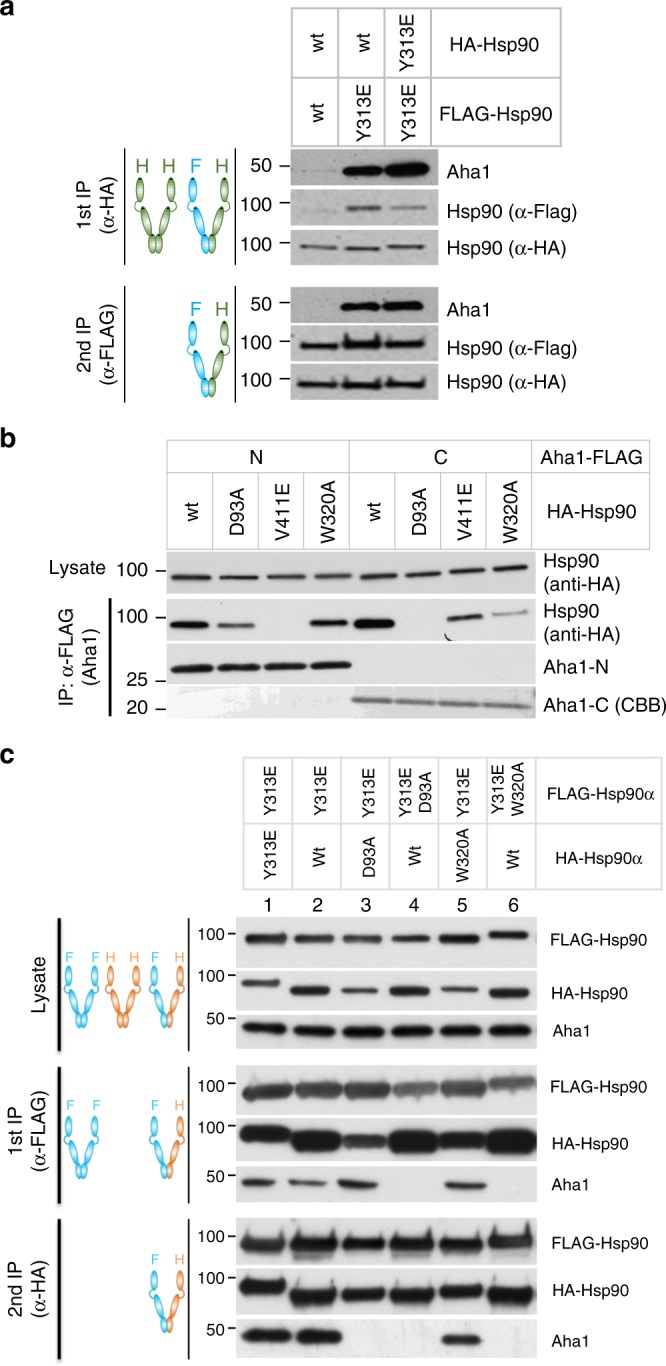
Enhanced Hsp90–Y313E–Aha1 interaction depends on Aha1-C binding to Hsp90-M domain. **a** Phosphomimetic substitution of Y313 in one protomer of Hsp90 is sufficient to stimulate Aha1 association. HA or FLAG tagged Hsp90, wild-type or Y313E, were co-expressed in 293A cells and sequentially immunoprecipitated with anti-HA and anti-FLAG antibody-linked resins. The first round of IP was performed with anti-HA antibody-linked resin; bound proteins were eluted with HA peptide and then loaded onto anti-FLAG antibody-linked resin. Proteins purified from the second round of IP were eluted with SDS-sample buffer and subjected to western blot analysis. Indicated proteins were detected with specific antibodies. **b** Hsp90 interaction with Aha1-N is abrogated by mutation in Hsp90-M while interaction with Aha1-C is compromised by mutation in both Hsp90-N and Hsp90-M. FLAG-tagged Aha1-N or Aha1-C was co-expressed with HA-tagged Hsp90, wild-type or with the indicated mutations. Aha1 domains were immunoprecipitated with mouse anti-FLAG antibody-linked resin, and Hsp90 was detected by western blot with rat anti-HA antibody. **c** FLAG or HA tagged Hsp90, wild-type or mutants, were co-expressed in 293A cells and sequentially immunoprecipitated and analyzed as in panel **a**. Y313E-stimulated association of full-length Aha1 with Hsp90 requires phosphomimetic mutation of Y313 on only one protomer, but requires ATP binding to Hsp90-N on both protomers, since D93A mutation on either protomer negates the impact of Y313E. Aha1 binding to Hsp90-Y313E is also disrupted by W320A mutation in cis (on the same protomer containing Y313E), but no disruption occurs when W320 mutation occurs in trans to Y313E. Source data for this figure are provided as a Source Data File

Hsp90 functions as a homodimer in cells. Thus, we investigated whether Y313 phosphorylation on both protomers is required to promote maximal Aha1 interaction. We co-expressed wild-type Hsp90 and Hsp90-Y313E with either FLAG or HA tag, and we performed sequential immunoprecipitations to isolate Hsp90 dimers with one protomer tagged with FLAG and the other tagged with HA (Fig. 8a). We found that the heterodimer wild-type Hsp90/Hsp90-Y313E and the homodimer Hsp90-Y313E/Hsp90-Y313E yielded a comparable amount of co-precipitated Aha1 (Fig. 8a, lower panel, lanes 2, 3). The increased Aha1 in lane 3 compared to lane 2 in the first-round immunoprecipitation is likely due to the additional presence of an Hsp90 dimer with both protomers tagged with HA, resulting in homodimers of Hsp90-Y313E/Hsp90-Y313E in lane 3 and homodimers of wild-type Hsp90/wild-type Hsp90 in lane 2 (Fig. 8a, upper panel). These data indicate that Y313 phosphorylation on only one protomer of the Hsp90 dimer is sufficient to promote enhanced Aha1 association (see also Fig. 8c, lanes 1 and 2).

To further define the interaction between Hsp90-M and Aha1-C that is enhanced by Y313 phosphorylation, we introduced a W320A point mutation in Hsp90 (see Supplementary Table 1). W320 is structurally close to Y313, and mutation of its homologous site in yeast Hsp90, W300, was reported to affect Aha1 association^30^. We first examined how W320A affected Hsp90 interaction with the individual domains of Aha1, and we found that it significantly reduced association of Aha1-C while minimally affecting Aha1-N interaction, suggesting that its impact is mechanistically distinct from that of V411E Hsp90 mutation, which disrupts Hsp90-M interaction with Aha1-N^6^ (Fig. 8b).

**Fig. 9:**
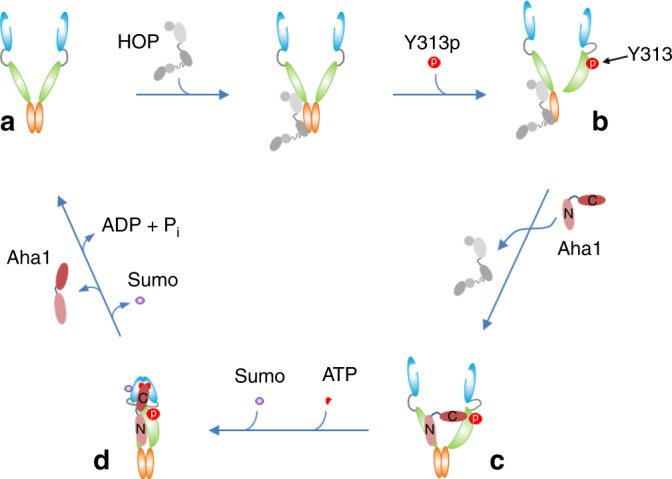
Y313 phosphorylation recruits Aha1 to Hsp90. The model shown here focuses on early steps in the cycle as Hsp90 proceeds from a client-loaded open conformation to dissociation of the client-loading co-chaperone p60^HOP^, and finally to recruitment of the ATPase-stimulating co-chaperone Aha1. An apo-Hsp90 dimer adopts the open conformation with the two N-domains un-engaged (**a**). Apo-Hsp90 can bind the co-chaperone p60^HOP^ to facilitate client transfer from Hsp70 (not shown in the cartoon) to Hsp90. Phosphorylation of Y313 in the Hsp90 middle domain induces a conformational change (**b**), weakening interaction with HOP and promoting recruitment of Aha1. Aha1 binds to the Hsp90 dimer while Hsp90 N-domains remain disassociated, with Aha1-N interacting with Hsp90-M of the un-phosphorylated Hsp90 protomer and Aha1-C interacting with Hsp90-M of the Hsp90 protomer bearing phosphorylated Y313 (**c**). Sumoylation of K191 in Hsp90-N in the presence of ATP^33^ promotes a repositioning of Aha1-C to engage the N-dimerized closed conformation that is required for activation of Hsp90 ATPase activity (**d**). Hydrolysis of ATP to ADP, and subsequent release of ADP, bias Hsp90 to re-occupy the open conformation, enabling it to re-enter the ATP-directed chaperone cycle

Next, we investigated the effect of introducing W320A either in cis or trans to Hsp90-Y313E. Sequential immunoprecipitations showed that W320A did not affect Aha1 association when it was in the protomer opposite to the one harboring Y313E (Fig. 8c, lane 5), but it completely disrupted Aha1 interaction when it was in the same protomer with Y313E (Fig. 8c, lane 6), confirming that W320 mutation functions in cis to disrupt Hsp90–Y313E interaction with Aha1-C. Taken together, these data support a model in which Y313 phosphorylation enhances interaction between Hsp90-M of the phosphorylated protomer with the C-domain of Aha1, while the Aha1 N-domain interacts with Hsp90-M of the opposite protomer (Fig. 9, step c).

Our preceding data suggest a model in which phosphomimetic substitution of Hsp90-Y313 initiates Aha1 association at an early step of the conformational cycle that is initiated upon Hsp90 binding a client protein. To determine whether N-domain ATP binding, in contrast to ATP-mediated N-domain dimerization, is necessary for stable association of Aha1 with either wild-type Hsp90 or Hsp90-Y313E, we first examined Hsp90-D93A (see Supplementary Table 1) association with the isolated N-domain and C-domain of Aha1. Hsp90α D93A mutation disrupts nucleotide binding to Hsp90-N^31^. We observed a moderate decrease in Aha1-N interaction with this Hsp90 mutant, suggesting either a subtle, long-range impact of the mutation itself on Hsp90-M conformation or an ATP-dependent effect on Aha1-N binding to Hsp90-M. At the same time, we observed a complete loss of interaction of Aha1-C, consistent with the prevailing model that some level of ATP-binding is required for stable Aha1-C interaction with Hsp90-N (Fig. 8b). We then examined the importance of ATP for Aha1 interaction with Hsp90-Y313E. Surprisingly, sequential immunoprecipitation showed that Hsp90-D93A mutation fully disrupted the enhanced Aha1 interaction promoted by Y313E phosphomimetic substitution, whether D93A was in the same or the opposite protomer as Y313E (Fig. 8c, lanes 3 and 4). These results indicate that nucleotide binding to both Hsp90 N-domains is necessary for stable interaction of Aha1 with Hsp90-Y313E, even if the direct effect of Y313 phosphomimetic mutation is to enhance formation of a transient Aha1/Hsp90 complex in which both domains of Aha1 interact independently with Hsp90-M. These data are consistent with a recent study demonstrating the cooperativity of nucleotide binding to both Hsp90 N-domains and its regulation by Aha1^32^.

## Discussion

Hsp90 exists as a homodimer and its chaperone activity requires traversal of a conformational cycle whose directionality is primarily dependent on ATP binding and hydrolysis. Binding of ATP biases Hsp90 towards closure of the two separated N-terminal domains, and hydrolysis of ATP, which requires N-domain dimerization, eventually leads to dissociation of the two N-domains. The ATPase activity of human Hsp90 is very modest and the most potent activator of this activity is the Hsp90 co-chaperone Aha1^7^. We previously reported that interaction between Aha1 and Hsp90 in cells is enhanced by phosphorylation on Y313 in the middle domain of Hsp90^16^. In this report, we explored this observation in more detail and we describe its mechanistic basis. We used the phosphomimetic glutamic acid substitution of Y313 to facilitate our study. This substitution does not compromise the overall structure of Hsp90 (see Supplementary Fig. 4c) and largely recapitulates the effects of Y313 phosphorylation, both in vitro and in vivo^16^ (also see Fig. 1a, b and Supplementary Table 3), while the equivalent mutation in yeast (Hsp82-Y293E) supports viability (see Supplementary Fig. 6). Further, Hsp90-Y313E retains basal ATPase activity and, compared to wild-type Hsp90, is more responsive to stimulation by Aha1^16^.

In vitro, association of Hsp90 with Aha1 is increased by ATP and AMPPNP; this has been interpreted as the result of enhanced interaction between Aha1-C and dimerized Hsp90-N domains^3,4^. Our results demonstrate that increased Hsp90–Aha1 interaction caused by Y313E phosphomimetic substitution is mediated by enhanced interaction of Aha1-C with Hsp90-M, but this intermediate complex still requires that the N-domain of each Hsp90 protomer have the ability to bind ATP. In contrast, however, the impact of this PTM on Aha1/Hsp90 interaction does not require the N-domain dimerized, closed conformation of Hsp90. Rather, it occurs at an early step after client loading, resulting in dissociation of p60^Hop^ and recruitment of Aha1, engendering a unique conformation in which the N-domains of each Hsp90 protomer remain disassociated. This hypothesis is supported by data derived from several orthogonal experiments. First, immunoprecipitation of Hsp90-Y313E yielded, in contrast to consistent and markedly increased association of Aha1, a clear and substantial decrease in association of p60^Hop^, a co-chaperone that associates with the N-domain undimerized conformation of Hsp90 and whose binding to Hsp90-M competes with that of Aha1. Second, while the nucleotide-stabilizing small molecule molybdate has a clear effect on Aha1 binding to wild-type Hsp90, it has a less dramatic, but additive impact on Aha1 binding to Hsp90-Y313E. Third, mutations that interfere with ATP-induced N–M domain repositioning (e.g., R400A and H210A), a prerequisite for N-domain dimerization, do not block Aha1 recruitment to Hsp90-Y313E. Fourth, E47A, an Hsp90 mutant that retains ATP binding but lacks ATPase activity, thus biasing Hsp90 towards an ATP-dependent closed conformation (with stronger affinity for Aha1), functions in an additive manner with Y313E to promote Aha1 association. Fifth, hydrogen exchange and NMR analyses show that the two N-domains of an Hsp90-Y313E homodimer are not dimerized in the absence of AMPPNP, but behave comparably to wild-type Hsp90 in the presence of this nucleotide. Sixth, NMR data show that addition of Aha1-C to Hsp90-Y313E results in prominent chemical shift changes within Hsp90-M that are not seen upon Aha1-C addition to wild-type Hsp90. Finally, NMR, ITC, and protein cross-linking data show that, in some respects, Hsp90 Y313 phosphomimetic mutation mimics the effect of AMPPNP on Hsp90 conformational dynamics and affinity for Aha1.

The extensive conformational impact of this phosphomimetic mutation on Hsp90 is likely due to the unique location of Y313. The conserved tyrosine localizes in Helix 11, packing against a β-sheet in Hsp90-M. The hydroxyl moiety of Y313 is engaged with the side chain of D319 via a hydrogen bond (PDB 3Q6M[10.2210/pdb3Q6M/pdb] and molecular modeling on PDB 2CG9[10.2210/pdb2CG9/pdb] and PDB 5FWM[10.2210/pdb5FWM/pdb]). Phosphorylation of Y313 would disrupt this interaction and is likely to significantly alter the conformational dynamics of Hsp90-M. Indeed, we detected significantly increased hydrogen exchange and broadening of NMR linewidths in the region surrounding Y313 for Hsp90-Y313E compared to wild-type Hsp90. Data from cross-linking experiments are also consistent with this possibility. The dynamic impact of Y313 phosphorylation is not limited to Hsp90-M, since conformational changes are clearly transmitted to both Hsp90 N-domain and C-domain as well. In addition, chemical shift changes observed for Ile residues at the interface of Hsp90 N-domain and M-domains indicate a unique reorientation of the two domains upon Y313 phosphorylation.

We have incorporated our current findings into an existing model^16,33,34^ of PTM-assisted Hsp90 cycling (Fig. 9). We identify Y313 on Hsp90 as a phosphorylation-sensitive conformational switch that initiates Aha1 recruitment to Hsp90, displacing p60^Hop^ after client loading and markedly enhancing Aha1-C affinity for Hsp90 while the chaperone is still in an open conformation. This initial interaction involves Aha1-N and Aha1-C domains binding to opposing surfaces on the middle domain of each Hsp90 protomer. Subsequently, ATP-dependent remodeling of the Hsp90-N and Hsp90-M domains, stabilized by Aha1-C binding to Hsp90-N, generates the closed conformation of the chaperone poised for ATP hydrolysis.

## Methods

### Cells, plasmids, and antibodies

HEK293 (Catalog number ATCC CRL-1573) cells were purchased from ATCC (Manassas, VA); 293A (Catalog number R70507) cells were purchased from Invitrogen (Carlsbad, CA). Both cells were grown in DMEM plus 10% fetal bovine serum. HA-tagged or FLAG-tagged Hsp90 sequence was cloned into pcDNA3 using the Bam HI/Xho I cutting sites, with the tag at the N-terminus of the Hsp90 sequence^35^. Plasmids expressing FLAG-tagged full length or truncated Aha1 proteins were a kind gift from Dr. Paul LaPointe (University of Alberta, Edmonton, Alberta, Canada)^3^. Point mutations were made by using the QuikChange site-directed mutagenesis method following the protocol of the company (Agilent, Santa Clara, CA). Hsp90 mutations used in this study are delineated in Supplementary Table 1. The forward primers used were as follows: Hsp90-E47A: GAGATCTTTCTGAGAGCGCTCATTTCAAATTCATCAGATGC; Hsp90-D93A:CTCTCACTATTGTGGCTACTGGAATTGGAATGAC; Hsp90-H210A: GGAGATTGTGAAGAAAGCTTCTCAGTTTATTGGATATCCC; Hsp90-Y313E:GGAGTACGGAGAATTCGAGAAGAGCTTGACCAATGAC; Hsp90-Y313F: GAGTACGGAGAATTCTTTAAGAGCTTGACCAATGA; Hsp90-W320A: GAGCTTGACCAATGACGCGGAAGATCACTTGGC; Hsp90-V391: CAACAAAGCAAAATTTTGAAAGAGATCAGGAAGAATTTGGTC. Each of these forward primers was paired with its reverse complement for making the mutant using the QuikChange method.

Mouse anti-FLAG antibody (M2)-linked (Catalog number A2220) and anti-HA antibody-linked (Catalog number A2095) resins, and rat monoclonal anti-HA antibodies (Catalog number 12158167001) were from Sigma-Aldrich (St. Louis, MO). Rabbit anti-Hsp90 (Catalog number 4877) and anti-Hop (Catalog number 5670) antibodies were purchased from Cell Signaling Technology (Beverly, MA). Polyclonal rabbit anti-FLAG antibody (Catalog number TA150078) was from ThermoFisher Scientific (Fremont, CA), and monoclonal mouse anti-FLAG antibody was from Sigma-Aldrich (Catalog number F3165). Rat anti-Hsp90 (Catalog number SPA-835), rabbit anti-Hsp90α (Catalog number SPS-771), mouse anti-Hsp90 (clone AC88), and anti-p23 polyclonal antibodies (Catalog number ADI-SPA-670) were from Enzo Life Sciences (Farmingdale, NY). Rabbit anti-Aha1 antibody (Catalog number 600-401-974) was from Rockland Immunochemicals (Limerick, PA). Site-specific rabbit anti-phosphoY313 polyclonal antibody was developed in collaboration with Cell Signaling Technology (Beverly, MA)^16^. The mouse anti-Hsp90 antibody H90-10 was a kind gift of Dr. Marc Cox (University of Texas at El Paso). Throughout this study, unless otherwise noted, we have used Hsp90 to designate human Hsp90α. Where relevant, measurements were taken from distinct samples.

### Immunoprecipitation and Western Blotting

Cultured HEK293 (ATCC, Manassas, VA) or 293A (Invitrogen, Carlsbad, CA) cells were transfected with Lipofectamine 2000 following the manufacturer’s protocol (ThermoFisher Scientific, Fremont, CA). Twenty-four hours after transfection, cells were washed once with PBS and lysed with Hepes buffer (20 mM Hepes, pH7.2, 100 mM NaCl, 1 mM MgCl_2_, and 0.5% Nonidet P40) containing complete protease inhibitors and phosSTOP phosphatase inhibitors (Sigma-Aldrich, St. Louis, MO). Lysis buffer was supplemented with a final concentration of 10 mM molybdate where indicated. Protein concentration was determined using a BCA Protein Assay Kit (ThermoFisher/Pierce Biotechnology, Rockford, IL). Immunoprecipitation was performed by incubating anti-FLAG antibody-linked or anti-HA antibody-linked resins with cleared cell lysate in microtubes. Resins were rotated at 4 °C for 1–2 h, washed three times with Hepes buffer with or without 10 mM molybdate, 1 mL per tube. Bound proteins were eluted with SDS-sample buffer (2% SDS, 10% glycerol, 0.1 M DTT, 0.002% bromophenol blue, and 0.08 M Tris–HCl pH6.8)^36^. For two-round immunoprecipitation, bound proteins on the resins used for the first round were eluted by incubating with Hepes buffer containing 100 µg/mL FLAG or HA peptide (Sigma-Aldrich) at 4 °C for 1 h. Eluate was loaded onto anti-HA or anti-FLAG antibody-linked resins for the second round immunoprecipitation, and bound proteins were eluted with SDS-sample buffer. Protein SDS–PAGE electrophoresis was performed using the Criterion system from Bio-Rad (Bio-Rad Laboratories, Hercules, CA), and the proteins in the gel were transferred to PVDF membrane following the protocol of the company. For western blotting, the membrane was first blocked with 2% non-fat milk in TNE buffer (10 mM Tris, pH 7.5, 50 mM NaCl, 2.5 mM EDTA, and 0.1% Tween 20). The primary antibodies were added to the blocking buffer and incubated overnight at 4 °C. The membrane was then washed three times with TNE buffer and incubated with horseradish peroxidase-linked secondary antibody at room temperature for 1 h. The membrane was washed again (four times) with TNE buffer, incubated with Pico ECL reagent from ThermoFisher Scientific (Rockford, IL), and exposed to HyBlot CL autoradiography film (Denville Scientific; Metuchen, NJ). Films were scanned with an Epson Perfection V700 scanner (Epson, Long Beach, CA), and the images were processed using Adobe Photoshop. All primary antibodies were used at a dilution of 1:1000; secondary antibodies were diluted 1:5000. Uncropped and processed blots and gels are provided in the Source Data file.

### Amide hydrogen exchange

Wild-type Hsp90 or Hsp90-Y313E (5 µl 60 µM) were pre-incubated in the absence of nucleotide for 5 min at 30 °C. Amide hydrogen exchange was initiated by diluting the sample 20-fold into D_2_O containing 25 mM Hepes/KOD pH 7.5, 10 mM KCl, and 5 mM MgCl_2_. After 30 s exchange at 30 °C, reactions were quenched by mixing the sample 1:1 with ice-cold quench buffer (400 mM KH_2_PO_4_/H_3_PO_4_ pH 2.2)^37,38^.

Quenched samples were injected directly into an HPLC-MS setup^38^ consisting of a nanoAcquity UPLC (Waters Corp.), a Shimadzu 10AD-VP HPLC pump, a Rheodyne 7725 manual injection valve with a 100 µl sample loop, a Microbore guard column with 2 mm inner diameter (IDEX, #C-130B) filled with pepsin (Sigma, #P6887) immobilized on Poros AL20 (Applied Biosystems, #1-6029-06), a six-port valve with microelectric actuator (Valco, #EPC6W), a microbore guard column with 1 mm inner diameter (IDEX, #C-128) filled with Poros R2 (Applied Biosystems #1-1118-02) as trap column, and an Acquity UPLC BEH C8 analytical column (Waters Corp. #186002876). Sample loop, trap column, and analytical column were submerged in an ice/water bath. The sample was pumped through the pepsin column onto the trap column at 200 µl min^−1^ (Shimadzu pump) and desalted for 2.5 min using ice-cold 0.1% formic acid in water. The peptic peptides were eluted from the trap column through the analytical column into the maXis QTOF mass spectrometer (Bruker Daltonics GmbH, Vienna, Austria) using a 10-min linear gradient of 90% ultrapure solvent A (0.1% formic acid in water)/10% ultrapure solvent B (0.1% formic acid in acetonitrile) to 45% solvent A/55% solvent B.

Data acquisition was performed on a maXis mass spectrometer (Bruker Daltonics GmbH) and analysis was performed using the Data Analysis software (Bruker Daltonics GmbH, Vienna, Austria)^38^. The identity of the peptides was determined by several independent MS/MS runs of identical samples in the absence of D_2_O, followed by MASCOT database searches. For the evaluation of deuteron incorporation the *m*/*z* and intensity values of each isotopic peak were manually extracted using the Data Analysis software and peptide centroid masses were determined using a custom Excel (Microsoft) sheet. The calculated centroid values were corrected for the back-exchange using a 100% deuterated sample prepared by denaturing Hsp90α in 6 M guanidinium/HCl and three cycles of lyophilizing the sample and re-dissolving it in D_2_O.

### Cross-linking of Hsp90 and Aha1

Purified protein concentrations were determined using the Bradford assay (Coomassie Protein Assay Kit, ThermoFisher/Pierce Biotechnology, Rockford, IL). 100 µg (1.18 nmol) of wild-type Hsp90 or Hsp90-Y313E dissolved in 50 µL of 25 mM HEPES pH 7.3, 100 mM NaCl were incubated with AMPPNP at 10 mM final concentration for 10 min on ice. Aha1-His was added to wild-type Hsp90α or Hsp90α-Y313E at an equimolar ratio and the mixture was incubated for 30 min on ice. Following incubation, the proteins were cross-linked by the addition of BDP-NHP cross-linker^39^ at a final concentration of 1 mM. The cross-linking reaction was allowed to proceed for 30 min at room temperature. After 30 min 50 µL of 0.1 M Tris buffer at pH 8.0 was added, followed by 100 µL of 8 M urea in 0.1 M Tris pH 8.0. Disulfide bonds were reduced with 5 mM TCEP for 30 min at room temperature, followed by alkylation with 10 mM iodoacetamide for 30 min at room temperature. The proteins were then digested using a 1:100 ratio of trypsin and incubating at 37 °C for 16 h. Following digestion, the resulting peptide mixture was desalted by solid phase extraction using a 50 mg C18 Sep-Pak cartridge (Waters, Milford, MA) and corresponding vacuum manifold to deliver solvents. The Sep-Pak cartridge was equilibrated by passing through 1 mL of acetonitrile (ACN) containing 0.1% trifluoroacetic acid (TFA), followed by 3 mL of H_2_O containing 0.1% TFA. The sample was acidified by adding TFA to a final concentration of 1% (v/v) and then passed through the Sep-Pak cartridge at a flow rate of 1 mL per minute. Excess salt was washed away by passing 5 mL of H_2_O containing 0.1% TFA through the cartridge. Peptides were eluted into a 2 mL Eppendorf tube by passing 1 mL of 80% ACN containing 0.1% TFA through the cartridge. The sample was then concentrated by vacuum centrifugation and adjusted to 100 µL final volume with 0.1% formic acid before LC–MS analysis.

### LC–MS analysis

Identification of cross-linked peptide pairs from the tryptic digest samples of cross-linked Hsp90 (wild-type/Y313E) and Aha1 was accomplished using a liquid chromatography–mass spectrometry (LC–MS) system comprised of a nano Acquity UPLC (Waters, Milford, MA) coupled to a Velos-FTICR mass spectrometer (Thermo Fisher Scientific, Grand Island, NY). The peptide samples were separated by reversed-phase chromatography using a 3 cm × 100 µm trapping column and a 60 cm × 75 µm analytical column both packed with 5 µm Reprosil C8 particles with 120 Å pores (Dr. Maisch HPLC GmbH, Ammerbuch, Germany). Peptides were loaded onto the trapping column using a flow rate 2 µL per minute to deliver an isocratic mobile phase composition of 98% solvent A (H_2_O containing 0.1% formic acid) and 2% solvent B (acetonitrile containing 0.1% formic acid) for 10 min. Reversed phase separation over the analytical column was performed by applying a linear gradient from 90% solvent A and 10% solvent B to 60% solvent A and 40% solvent B over 120 min at a flow rate of 300 nL per minute. Eluting peptides were ionized by electrospray ionization by applying a voltage of 2.2 kV to a laser-pulled tip at the tip of the analytical column. The Velos-FTICR mass spectrometer was operated using a real-time analysis for cross-linked peptide technology (ReACT) acquisition method 30. A MS1 scan was performed in the ICR cell at a resolving power of 50,000 using an automatic gain control (AGC) target value of 5E5 ions. The most intense precursor ion with a charge state of ≥4 was isolated in the ion trap with a 3*m/z* window, fragmented by collision-induced dissociation with a normalized collision energy setting of 25 and the fragment ions measured by MS2 analysis in the ICR cell with a resolving power setting of 50,000 and an AGC value of 2E5. The ReACT method performed a check for ions detected in the MS2 scan that satisfy the expected mass relationship for cross-linked peptide pairs (mass precursor ion = mass peptide 1 + mass peptide 2 + reporter ion) within a 20 ppm mass error tolerance. Released peptide ions that satisfy the relationship were subjected to MS3 analysis in the ion trap analyzer using an isolation window of 3*m/z*, a normalized collision energy of 35 and an AGC target value of 5E4. Targeted quantitative analysis of 22 selected cross-linked peptide pairs was accomplished by PRM. Reversed-phase chromatographic separation of cross-linked peptide samples was performed using an EASY-nLC 1000 system (Thermo Fisher Scientific, Grand Island, NY) equipped with a 3 cm × 100 µm trapping column and a 60 cm × 75 µm analytical column both packed with 5 µm Reprosil C8 particles with 120 Å pores (Dr. Maisch HPLC GmbH, Ammerbuch, Germany). Peptides were loaded onto the trapping column using 20 µL of solvent A (H_2_O containing 0.1% formic acid) at a flow rate 2 µL per minute. Reversed phase separation over the analytical column was performed by applying a linear gradient from 98% solvent A and 2% solvent B (acetonitrile containing 0.1% formic acid) to 60% solvent A and 40% solvent B over 120 min at a flow rate of 300 nL per minute. Eluting peptides were ionized by electrospray ionization by applying a voltage of 2.2 kV to a laser pulled tip at the tip of the analytical column. Mass spectrometry was performed using Q-Exactive Plus mass spectrometer (Thermo Fisher Scientific, Grand Island, NY) operated using a PRM method with the following settings: a resolving power of 17,500@200*m/z*, AGC target of 2E5 ions, maximum ion time of 100 ms, isolation window of 3*m/z* and a normalized collision energy of 27.

### Data analysis

Assignment of cross-linked peptide sequences was performed by using Comet v. 2018.01.2^40^ to search the data generated from the ReACT LC–MS experiments. Comet settings included: searching a database comprised of forward and reverse Hsp90 (wild-type and Y313) and Aha1 protein sequences, a precursor mass tolerance of 20 ppm allowing for −1, 0, 1, 2, or 3 ^13^C offsets, allowing for only fully tryptic peptide sequences with up to three missed cleavage sites, up to two occurrences per peptide of oxidation of Met (15.9949 Da) as a variable modification and a single occurrence of the BDP cross-linker residual mass (197.032422 Da) on Lys as a required modification, carbamidomethylation of Cys (57.021464 Da) as a fixed modification, fragment ion tolerance of 1.0005 Da with a bin offset of 0.4, and a digest mass range from 600 to 5000 Da. The Comet search resulted in a total of 162 non-redundant fully assigned cross-linked peptide pairs, including 147 target sequences and 15 decoys (peptide pair containing at least one reverse sequence peptide) for a maximum estimated false discovery rate (FDR) of 10.2% at the non-redundant peptide pair level (Supplementary Data 1). From this set of 147 target peptide pairs, 22 confidently (maximum estimated FDR 2.2%) assigned cross-linked peptide pairs, including five inter-protein links between Hsp90 and Aha1, were selected for targeted quantitative PRM analysis.

Mass spectrometry data from the PRM experiments were analyzed using Skyline (v. 3.5.0.9319)^39^. Transitions for cross-linked peptides were imported into Skyline using the Cross-link Transition Calculator external tool (https://skyline.ms/skyts/home/software/Skyline/tools/details.view?name=Cross-link%20Transition%20Calculator). Integrated peak areas were exported from Skyline (Supplementary Fig. 3b, c) and used to calculate the log2(AMPPNP/Apo) ratios for each cross-linked peptide pair. Normalization across samples was accomplished by starting with equivalent amount of protein, 1.18 nmol of Hsp90 (wild-type or Y313E) and Aha1 as described in the cross-linking section of the methods above. Statistical significance was determined by calculation of the 95% confidence intervals (*n* = 6 independent experiments) for the mean integrated peak areas, as well as the mean log2(AMPPNP/Apo) values.

### NMR spectroscopy

^1^H-^13^C HMQC spectra (methyl-TROSY) of ^2^H/^13^C Ile-labeled Hsp90 were acquired at 25 °C on an Agilent dd800 MHz instrument equipped with a cryoprobe. Sample concentration for wild-type and Y313E Hsp90 ranged between 85 and 110 mM Hsp90, while the concentration of the ^2^H-labeled partner (full-length Aha1, Aha1-N, or Aha1-C) was kept at half the Hsp90 concentration. When present, the concentration of AMPPNP was kept constant at 2.5 with 5 mM MgSO_4_. All spectra were acquired in 20 mM deuterated Tris pH 7.5, 100 mM NaCl, 0.5 mM EDTA, 1.5 mM DTT, and in 100% D_2_O. The assignment performed by Dyson and coworkers was used^41^.

### Isothermal titration calorimetry

For the data displayed in Supplementary Table 3, titrations were carried out on a PEAQ-ITC calorimeter (Malvern Scientific, Malvern, UK) at 36 °C, in 20 mM Tris pH 7.5, 100 mM NaCl, 0.5 mM EDTA, 5 mM MgSO_4_, and 1 mM Tris(2-carboxyethyl)phosphine. When indicated, AMPPNP was included in both the cell and the syringe at a concentration of 2 mM. The 200 μL sample cell was filled with Hsp90 at a concentration of 30 μM, and the 40 μL injection syringe was filled with Aha1 constructs at a concentration of 380–450 μM. All experiments were performed with an initial 0.2 μL injection followed by twelve 3 μL injections, with a 4 min time interval between each injection. Isotherms were processed using Origin 7.0, excluding the initial injection. All titrations were performed in three technical repeats. The data presented in Supplementary Table 2 were obtained separately and the ITC buffer contained 20 mM Tris pH 7.5, 5 mM NaCl, 1 mM EDTA, and the temperature was 30 °C.

### Analytical size exclusion chromatography (SEC)

Frozen aliquots of wild-type Hsp90 and Hsp90-Y313E in 20 mM Tris pH 7.5, 500 mM NaCl, 2 mM EDTA and 5 mM 2-mercaptoethanol, at a concentration of ~5 mg/mL, were thawed and exchanged in SEC-buffer (20 mM Tris pH 7.5, 100 mM NaCl, 0.5 mM EDTA, 1.5 mM DTT). Subsequently, proteins were concentrated to ~25 μM, and a 150 μL sample was injected into an ENrichSEC650 10 × 300 column (Bio-Rad, Hercules, CA) pre-equilibrated with SEC-buffer. Runs were performed at 4 °C, at a flow rate of 0.75 mL/min.

### Reporting summary

Further information on research design is available in the Nature Research Reporting Summary linked to this article.

## Supplementary information

Supplementary Information

Description of Additional Supplementary Files

Supplementary Data 1

Reporting Summary

Source Data

## Data Availability

The cross-linking mass spectrometry data have been deposited to the ProteomeXchange Consortium via the PRIDE partner repository with the dataset identifier PXD013476. The source data underlying Figs. 1, 2b–e, 3, 7, 8, Supplementary Figs. 2a, 3a, 3b, 3c, 6b, and Supplementary Tables 2, and 3 are provided as a Source Data file. A reporting summary for this Article is available as a Supplementary Information file. The mean values used to calculate the HDX-MS data in Fig. 3 are provided in the Source Data file; the data underlying these means and the raw mass spectrometric data are no longer available. All other data supporting the findings of this study are available from the corresponding author upon reasonable request.

## References

[1] Xu W, Neckers L (2007). Targeting the molecular chaperone heat shock protein 90 provides a multifaceted effect on diverse cell signaling pathways of cancer cells. Clin. Cancer Res..

[2] Li J, Buchner J (2013). Structure, function and regulation of the hsp90 machinery. Biomed. J..

[3] Koulov AV (2010). Biological and structural basis for Aha1 regulation of Hsp90 ATPase activity in maintaining proteostasis in the human disease cystic fibrosis. Mol. Biol. Cell.

[4] Retzlaff M (2010). Asymmetric activation of the hsp90 dimer by its cochaperone aha1. Mol. Cell.

[5] Daturpalli S, Kniess RA, Lee CT, Mayer MP (2017). Large rotation of the N-terminal domain of Hsp90 is important for interaction with some but not all client proteins. J. Mol. Biol..

[6] Meyer P (2004). Structural basis for recruitment of the ATPase activator Aha1 to the Hsp90 chaperone machinery. Embo J..

[7] Panaretou B (2002). Activation of the ATPase activity of hsp90 by the stress-regulated cochaperone aha1. Mol. Cell.

[8] Schulze A (2016). Cooperation of local motions in the Hsp90 molecular chaperone ATPase mechanism. Nat. Chem. Biol..

[9] Zierer BK (2016). Importance of cycle timing for the function of the molecular chaperone Hsp90. Nat. Struct. Mol. Biol..

[10] Mollapour M, Neckers L (2012). Post-translational modifications of Hsp90 and their contributions to chaperone regulation. Biochim Biophys. Acta.

[11] Prodromou C (2017). Regulatory mechanisms of Hsp90. Biochem Mol. Biol. J..

[12] Woodford MR (2016). Impact of posttranslational modifications on the anticancer activity of Hsp90 inhibitors. Adv. Cancer Res.

[13] Soroka J (2012). Conformational switching of the molecular chaperone Hsp90 via regulated phosphorylation. Mol. Cell.

[14] Stetz G, Tse A, Verkhivker GM (2018). Dissecting structure-encoded determinants of allosteric cross-talk between post-translational modification sites in the Hsp90 chaperones. Sci. Rep..

[15] Bachman AB (2018). Phosphorylation induced cochaperone unfolding promotes kinase recruitment and client class-specific Hsp90 phosphorylation. Nat. Commun..

[16] Xu W (2012). Dynamic tyrosine phosphorylation modulates cycling of the HSP90-P50(CDC37)-AHA1 chaperone machine. Mol. Cell.

[17] Muller P, Ceskova P, Vojtesek B (2005). Hsp90 is essential for restoring cellular functions of temperature-sensitive p53 mutant protein but not for stabilization and activation of wild-type p53: implications for cancer therapy. J. Biol. Chem..

[18] Barent RL (1998). Analysis of FKBP51/FKBP52 chimeras and mutants for Hsp90 binding and association with progesterone receptor complexes. Mol. Endocrinol..

[19] Graf C, Lee CT, Eva Meier-Andrejszki L, Nguyen MT, Mayer MP (2014). Differences in conformational dynamics within the Hsp90 chaperone family reveal mechanistic insights. Front Mol. Biosci..

[20] Rehn AB, Buchner J (2015). p23 and Aha1. Sub-Cell. Biochem..

[21] Karagoz GE (2011). N-terminal domain of human Hsp90 triggers binding to the cochaperone p23. Proc. Natl Acad. Sci. USA.

[22] Martinez-Yamout MA (2006). Localization of sites of interaction between p23 and Hsp90 in solution. J. Biol. Chem..

[23] Ratzke C, Hellenkamp B, Hugel T (2014). Four-colour FRET reveals directionality in the Hsp90 multicomponent machinery. Nat. Commun..

[24] Southworth DR, Agard DA (2008). Species-dependent ensembles of conserved conformational states define the Hsp90 chaperone ATPase cycle. Mol. Cell.

[25] Sullivan W (1997). Nucleotides and two functional states of hsp90. J. Biol. Chem..

[26] Sullivan WP, Owen BA, Toft DO (2002). The influence of ATP and p23 on the conformation of hsp90. J. Biol. Chem..

[27] Cunningham CN, Southworth DR, Krukenberg KA, Agard DA (2012). The conserved arginine 380 of Hsp90 is not a catalytic residue, but stabilizes the closed conformation required for ATP hydrolysis. Protein Sci..

[28] Grenert JP, Johnson BD, Toft DO (1999). The importance of ATP binding and hydrolysis by hsp90 in formation and function of protein heterocomplexes. J. Biol. Chem..

[29] Lanman J, Prevelige PE (2004). High-sensitivity mass spectrometry for imaging subunit interactions: hydrogen/deuterium exchange. Curr. Opin. Struct. Biol..

[30] Hawle P (2006). The middle domain of Hsp90 acts as a discriminator between different types of client proteins. Mol. Cell Biol..

[31] Obermann WM, Sondermann H, Russo AA, Pavletich NP, Hartl FU (1998). In vivo function of Hsp90 is dependent on ATP binding and ATP hydrolysis. J. Cell Biol..

[32] Wortmann P, Gotz M, Hugel T (2017). Cooperative nucleotide binding in Hsp90 and its regulation by Aha1. Biophys. J..

[33] Mollapour M (2014). Asymmetric Hsp90 N domain SUMOylation recruits Aha1 and ATP-competitive inhibitors. Mol. Cell.

[34] Wolmarans Annemarie, Kwantes Allison, LaPointe Paul (2019). A novel method for site-specific chemical SUMOylation: SUMOylation of Hsp90 modulates co-chaperone binding in vitro. Biological Chemistry.

[35] Scroggins BT (2007). An acetylation site in the middle domain of Hsp90 regulates chaperone function. Mol. Cell.

[36] Xu W (2001). Sensitivity of mature Erbb2 to geldanamycin is conferred by its kinase domain and is mediated by the chaperone protein Hsp90. J. Biol. Chem..

[37] Rist W, Graf C, Bukau B, Mayer MP (2006). Amide hydrogen exchange reveals conformational changes in hsp70 chaperones important for allosteric regulation. J. Biol. Chem..

[38] Hentze, N., Mayer, M. P. Analyzing protein dynamics using hydrogen exchange mass spectrometry. *J. Vis. Exp.* **81**, e50839. 10.3791/50839 (2013). 10.3791/50839 PMC3992118 24326301

[39] Chavez JD, Schweppe DK, Eng JK, Bruce JE (2016). In vivo conformational dynamics of Hsp90 and its interactors. Cell Chem. Biol..

[40] Eng JK, Jahan TA, Hoopmann MR (2013). Comet: an open-source MS/MS sequence database search tool. Proteomics.

[41] Park SJ, Kostic M, Dyson HJ (2011). Dynamic interaction of Hsp90 with its client protein p53. J. Mol. Biol..

