# Supplementary material for: Hsp90 middle domain phosphorylation initiates a complex conformational program to recruit the ATPase-stimulating cochaperone Aha1
Source: Nat Commun. 2019 Jun 12;10:2574. doi: 10.1038/s41467-019-10463-y (PMC6561935; doi:10.1038/s41467-019-10463-y)
Supplement: Supplementary file 1 — Supplementary Information [file 41467_2019_10463_MOESM1_ESM.pdf]

Hsp90 middle domain phosphorylation initiates a complex conformational program to recruit the ATPase-stimulating cochaperone Aha1

Xu et al.

Supplementary Information

Supplementary Figures, Tables and References

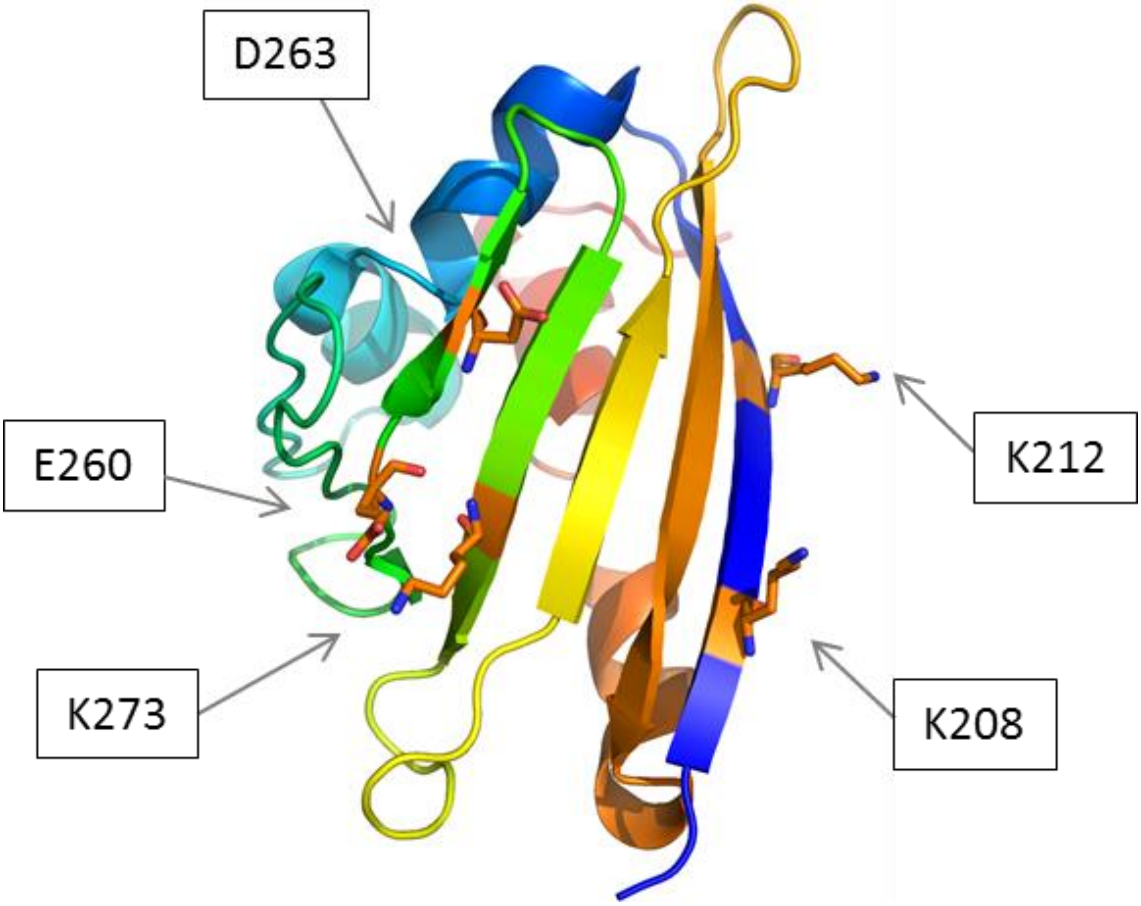

Supplementary Figure 1. The structure of Aha1-C (PDB 1X53), with residues that are mutated in Figure 1E labeled and shown in stick form.

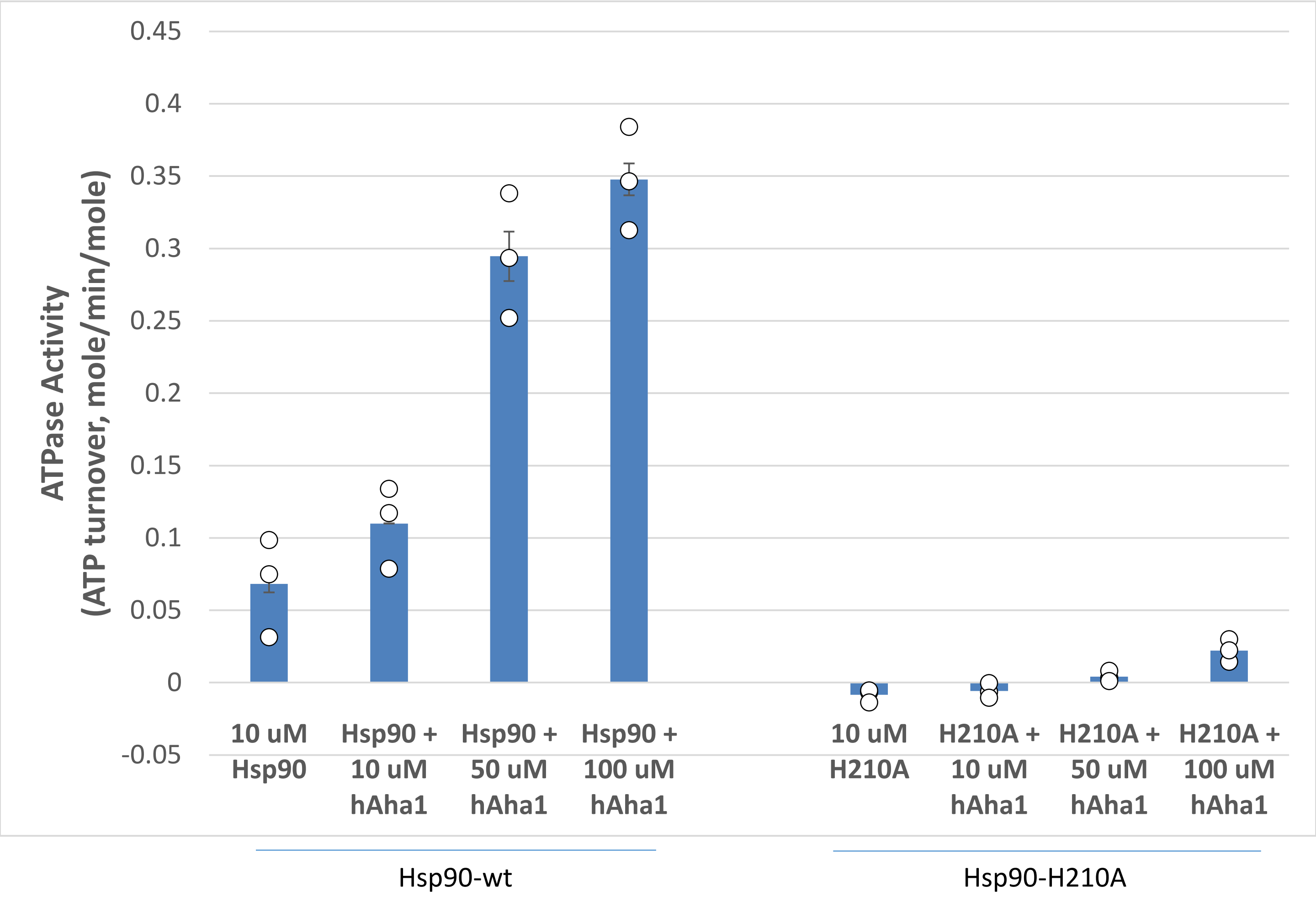

Supplementary Figure 2a. H210A mutation abrogates both the basal and Aha1-stimulated ATPase activity of Hsp90. Basal ATPase activity (1st column in each set) of purified wild-type Hsp90 and Hsp90-H210A proteins (10μM) was measured in vitro using a coupled enzyme assay that included an ATP-regenerating system [1]. Hsp90-specific ATPase activity was determined by subtracting the non-specific activity remaining in the presence of the Hsp90 inhibitor geldanamycin (60 μM). The impact of adding increasing concentrations of human Aha1 (hAha1) on Hsp90-specific ATPase activity is also shown. Data are expressed as moles ATP hydrolyzed/min/mole Hsp90. Experiments were performed a minimum of 3 times; data are expressed as mean +/- S.D.

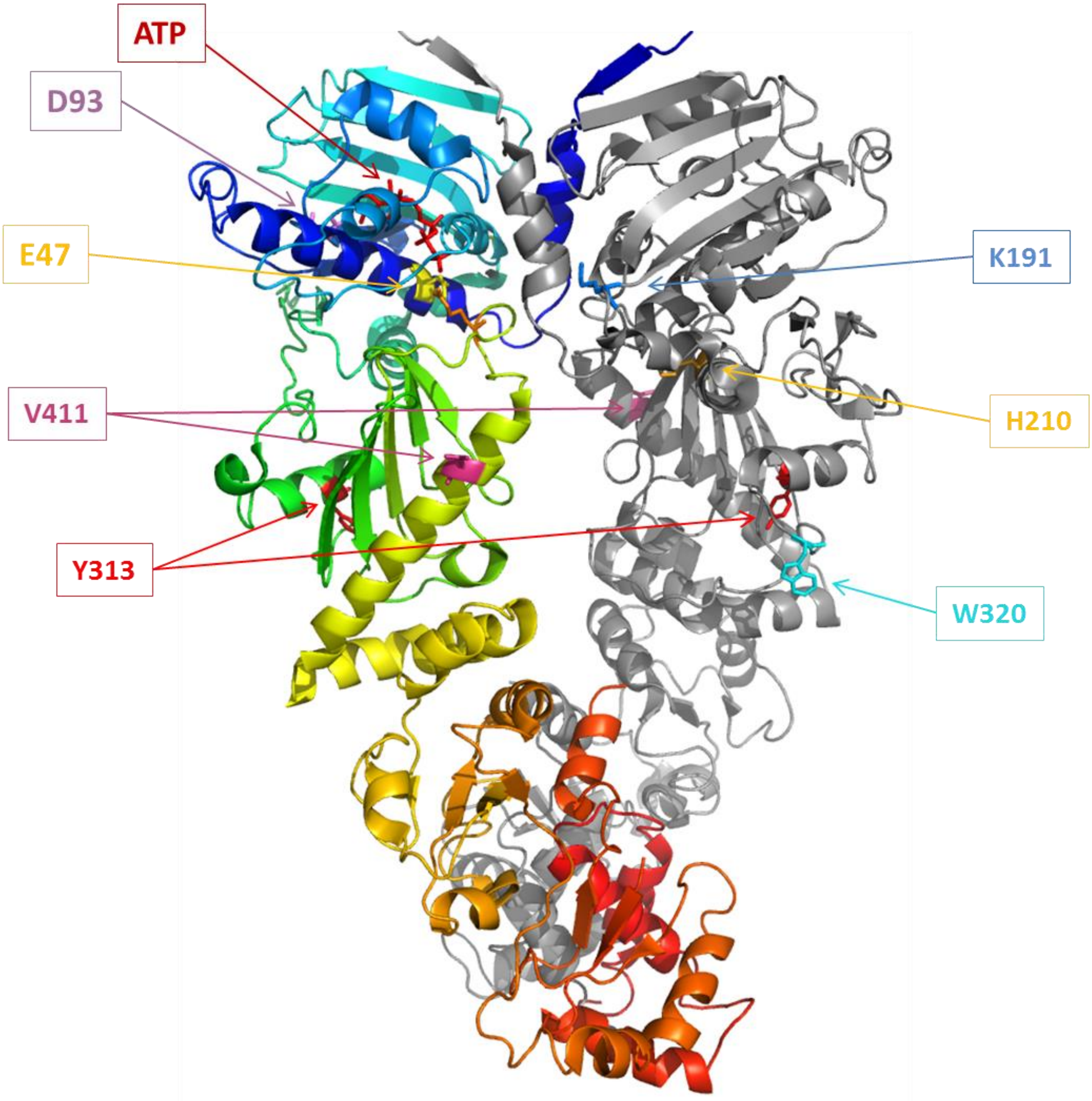

Supplementary Figure 2b. Human Hsp90α structure modeled on that of yeast Hsp90 (2CG9). One protomer is in grey while the other is in color, with the residues mutated in this manuscript K191 being the exception) highlighted. K191 is the sumolylated N-domain lysine residue that, when mutated to arginine (non-sumoylatable) abrogates Aha1 association (previously published, see reference 3). Source data for this figure are provided as a Source Data File.

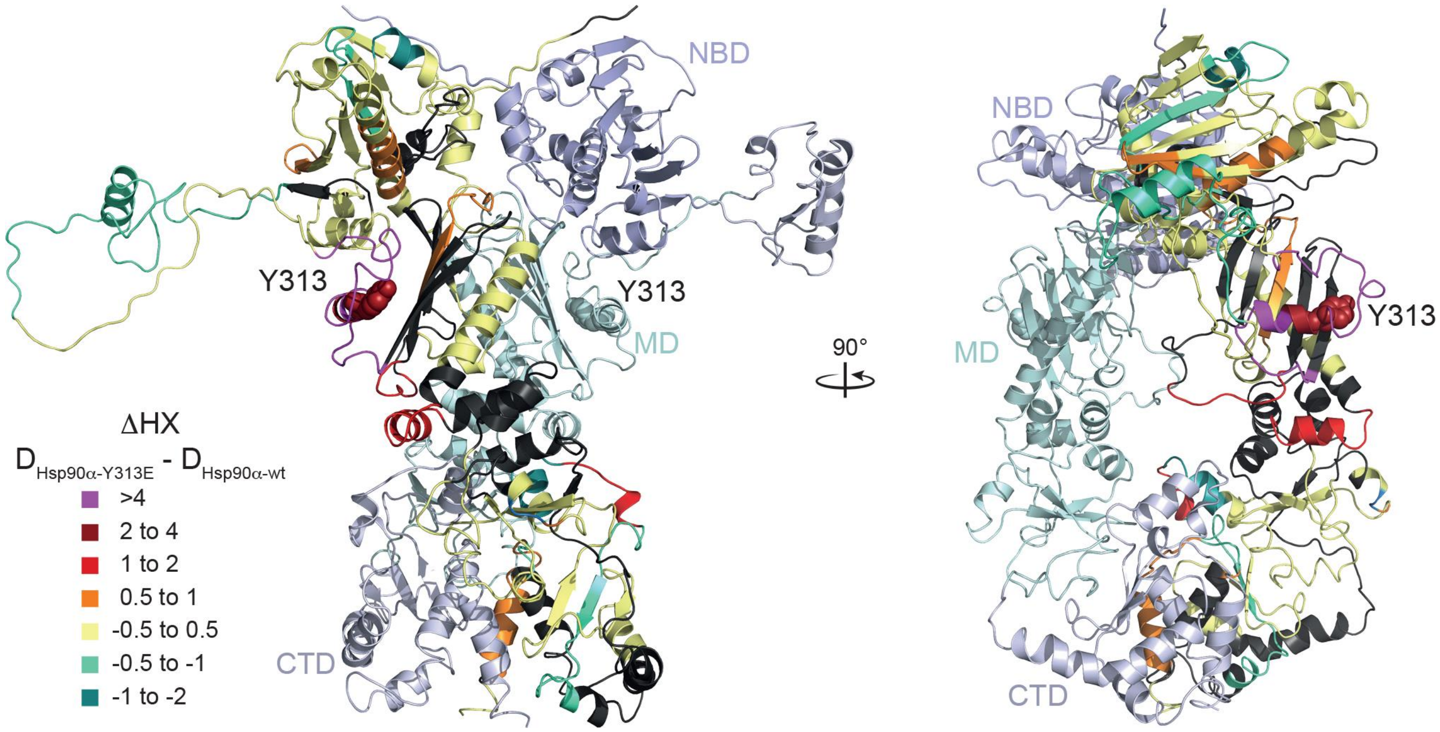

Supplementary Figure 3a. Homology model of human Hsp90α structure based on that of yeast Hsp90 (2CG9). One protomer is colored light blue, while the other is colored according to the differences between Hsp90-Y313E and wild-type Hsp90 in hydrogen exchange.

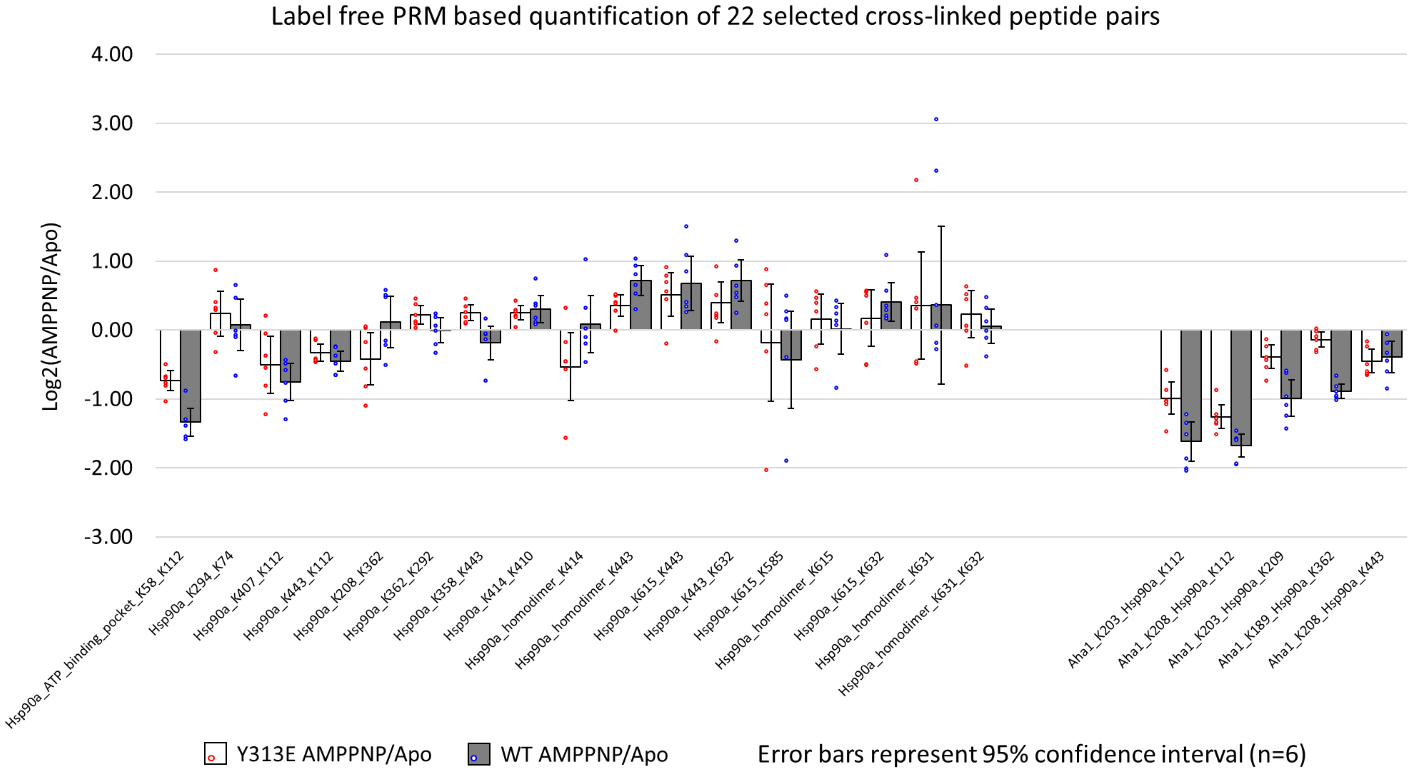

Supplementary Figure 3b. Phosphomimetic substitution of Y313 affects Hsp90 conformation and Hsp90-Aha1 interaction. Purified Hsp90 (wild-type or Y313E) and Aha1 proteins were mixed in the presence or absence of AMPPNP, followed by the addition of the crosslinking reagent BDP-NHP which crosslinks adjacent lysines. Samples were then subjected to LC-MS analysis; crosslinked lysines were identified and crosslink intensities were calculated and compared between wild-type and Y313E Hsp90. Error bars represent 95% confidence interval (n=6).

Label free PRM based quantification of
17 selected cross-linked peptide pairs of Hsp90 protein

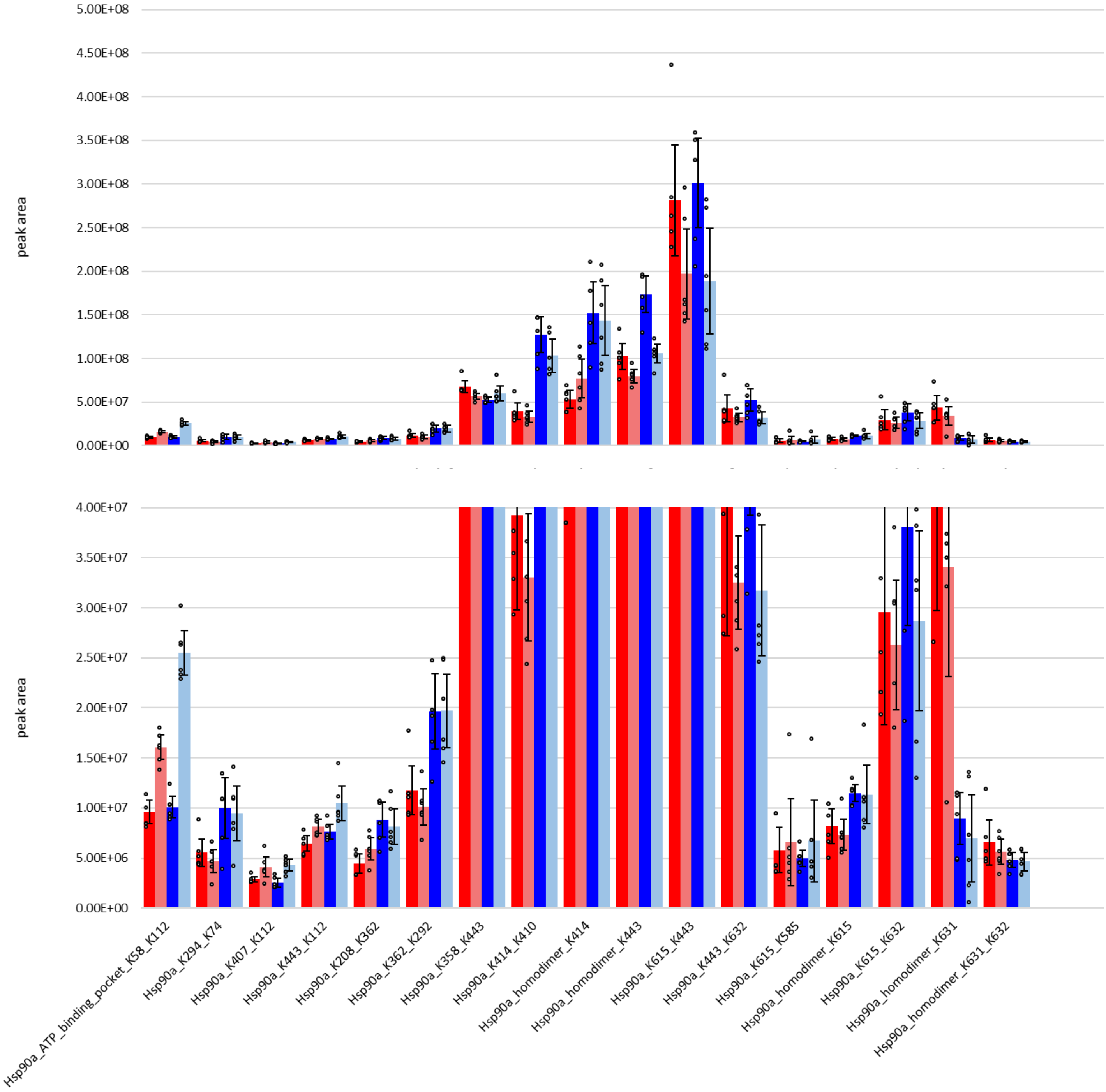

Supplementary Figure 3c. Experiments were performed as described in Figure 7, except that the data are label free PRM based quantification of crosslinked peptides of Hsp90. Error bars represent 95% confidence interval (n=6). Source data for this figure are provided as a Source Data File.

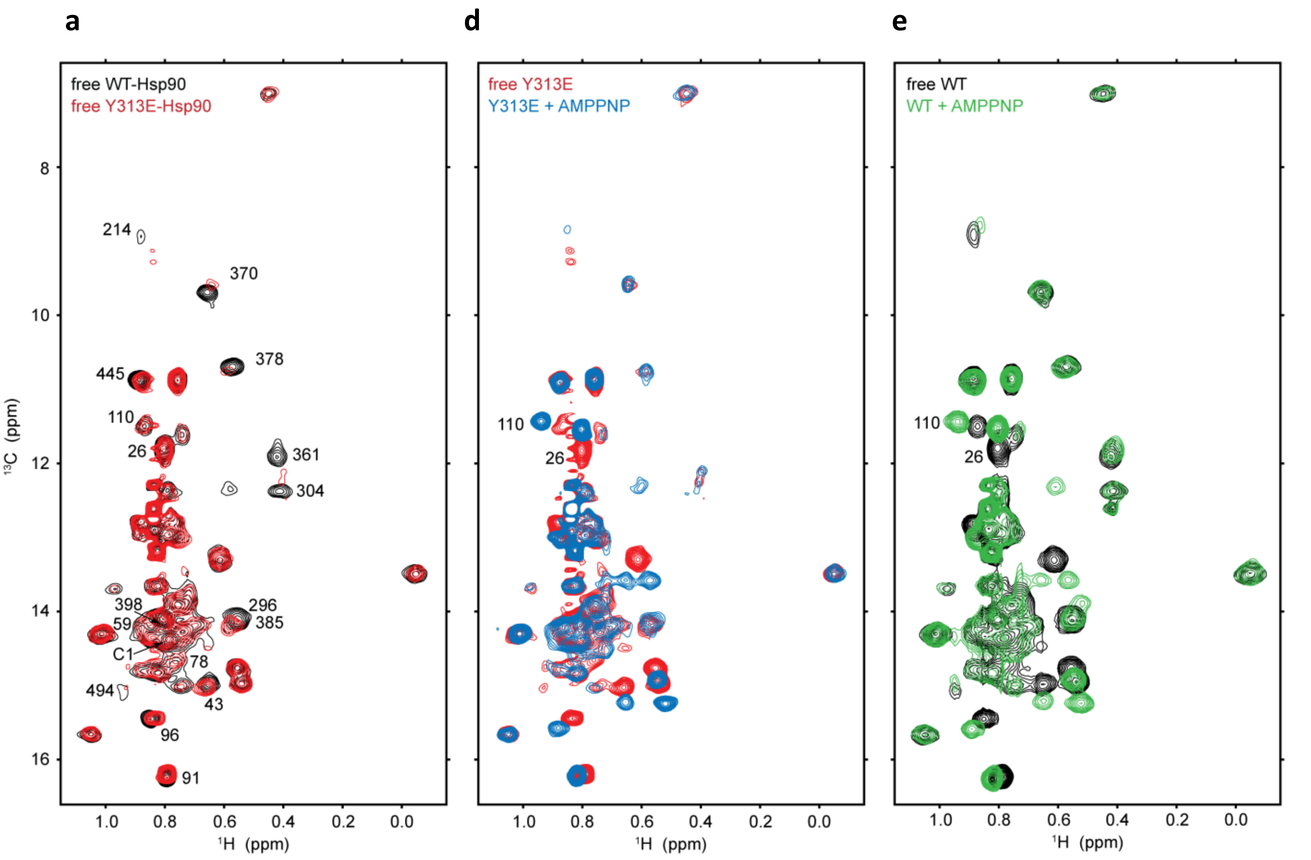

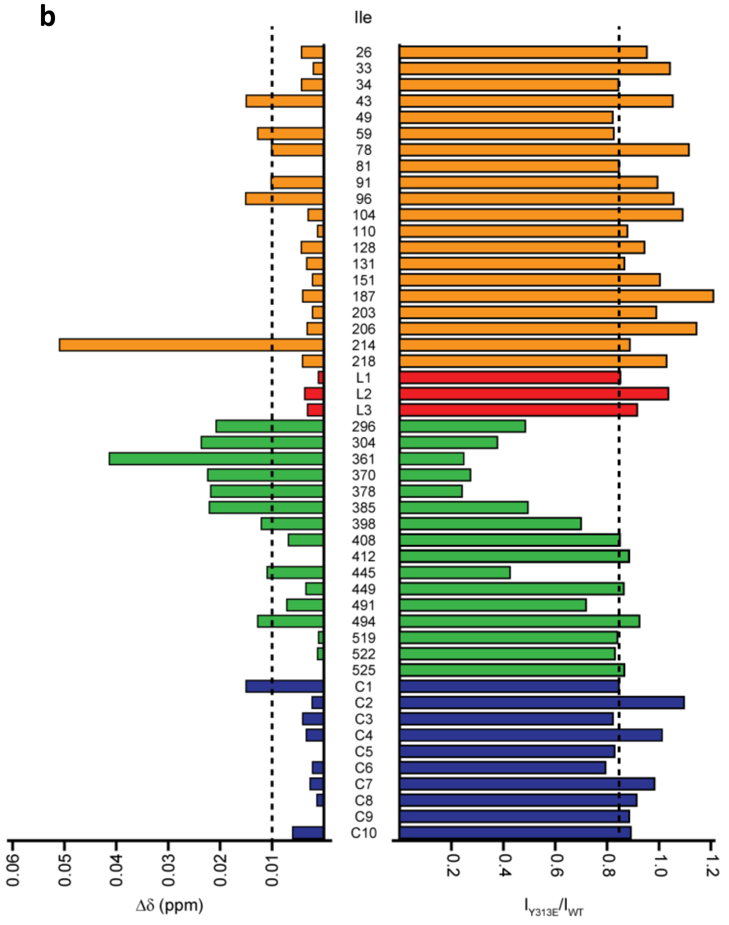

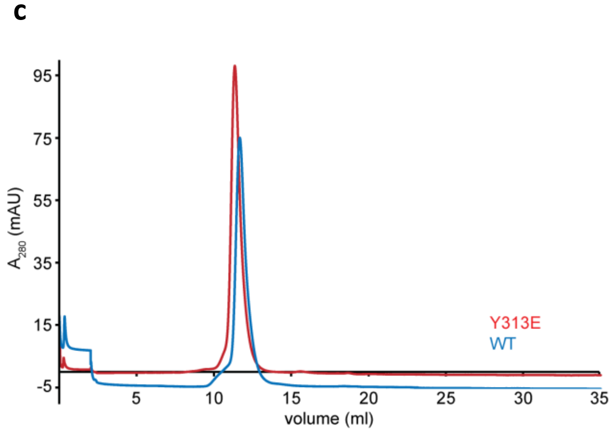

	^{1}H linewidth (Hz)		
	**WT**	**Y313E**	
I43	38.0	36.6	Hsp90-N
I91	27.3	29.3	
I96	30.1	27.4	
I304	30.5	184.0	Hsp90-M
I361	51.0	192.0	
I370	31.1	67.7	
I378	30.8	52.2	
I522	28.7	31.1	
C2	36.8	40.5	Hsp90-C
C6	34.6	37.1	

Supplementary Figure 4. Comparison of the ^1H-^{13}C-HMQC spectra of Y313E and wild-type Hsp90 in the free- and AMPPNP-bound states. **a.** Overlay of the full ^1H-^{13}C-HMQC spectra of wild-type (black) and Y313E-Hsp90 (red), showing the assignment of the Ile signals that experience changes in chemical shift and line broadening as a result of the phosphomimetic mutation. **b.** The effect of the phosphomimetic mutation on the chemical shift and linewidths of Hsp90 Ile signals shown as a normalized chemical shift difference, $\Delta\delta$, (left) and intensity ratio plots (right). Dotted lines mark average changes, which is taken as a significance threshold. Signals from N-, M-, C-domains and the linker, are colored in orange, green, blue and red, respectively. **c.** The phosphomimetic mutation does not result in Hsp90 aggregation. Top: elution profile of wild-type (blue) and Y313E (red) Hsp90 through an analytical gel-filtration column. Bottom: Comparison of signal linewidths for a representative set of Ile residues from all three domains. In agreement with the intensity ratios shown in b, only Hsp90-M Ile signals show altered linewidths. **d.** The spectrum of AMPPNP-bound Hsp90-Y313E (blue) mapped on the spectrum of free Hsp90-Y313E (red). **e.** The spectrum of AMPPNP-bound wild-type Hsp90 (green) mapped on the spectrum of free wild-type Hsp90 (black). The signals of I110 and I26 that are sensitive to Hsp90-N dimerization are highlighted. When compared to the effect of AMPPNP binding to wild-type Hsp90, Y313E does not induce a closed N-/N-dimerized state as evident by the lack of characteristic chemical shift changes, such as for the signals of I110 or I26, which lie at the inter-protomer interface in the closed state. However, binding of AMPPNP to Y313E induces the same Hsp90-N/N-dimerized state observed upon AMPPNP binding to wild-type Hsp90, demonstrating that Y313 phosphomimetic mutation does not interfere with nucleotide-induced N-domain dimerization. Source data for this figure are provided as a Source Data File.

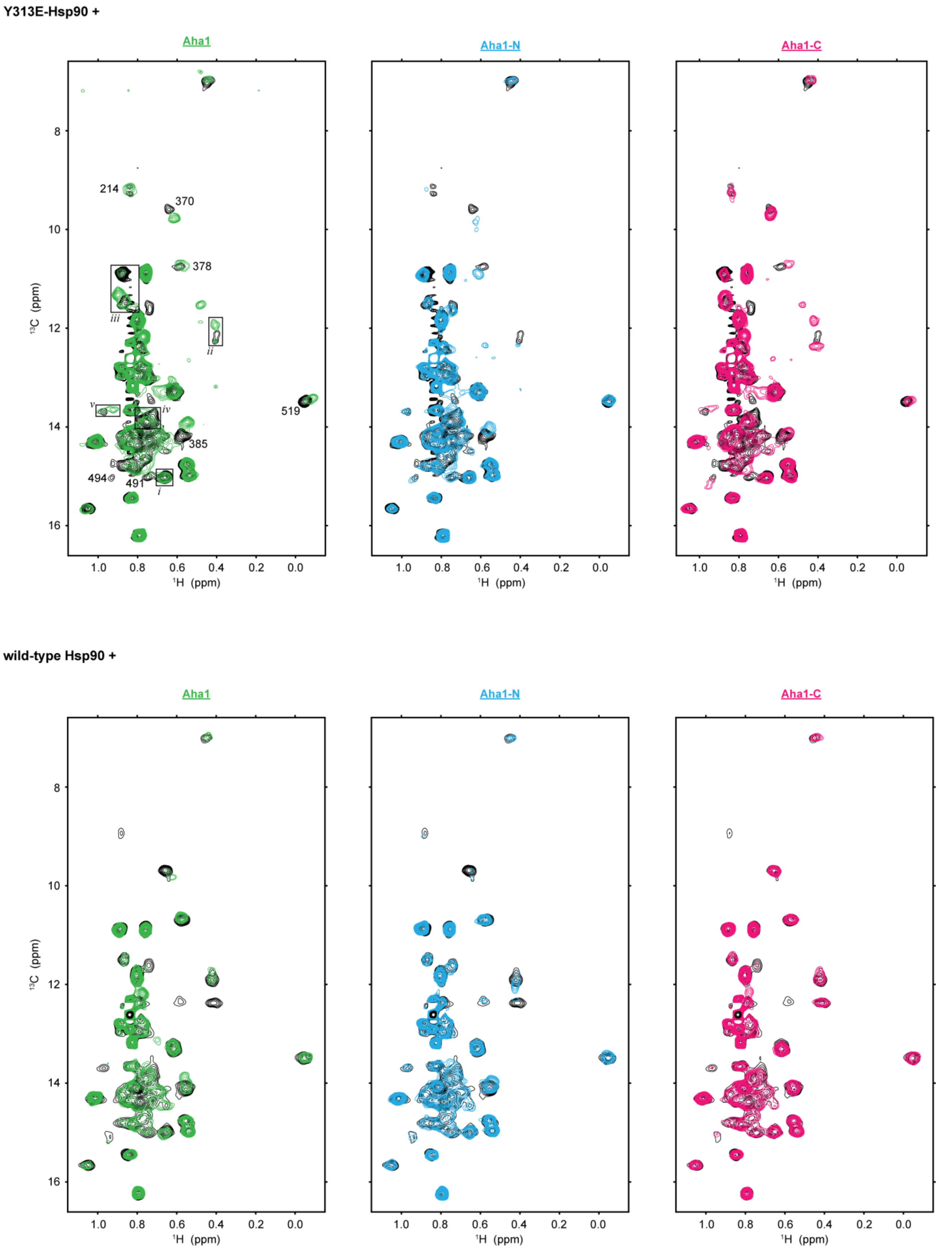

Supplementary Figure 5. ^{1}H-^{13}C HMQC titrations of Y313E (top) and wild-type Hsp90 (bottom), with Aha1 (green), Aha1-N (blue) and Aha1-C (pink). Boxed regions with roman numbering refer to expansions presented in Figure 5 of main text. Other relevant signals and assignments omitted from Figure 5, is provided. Source data for this figure are provided as a Source Data File.

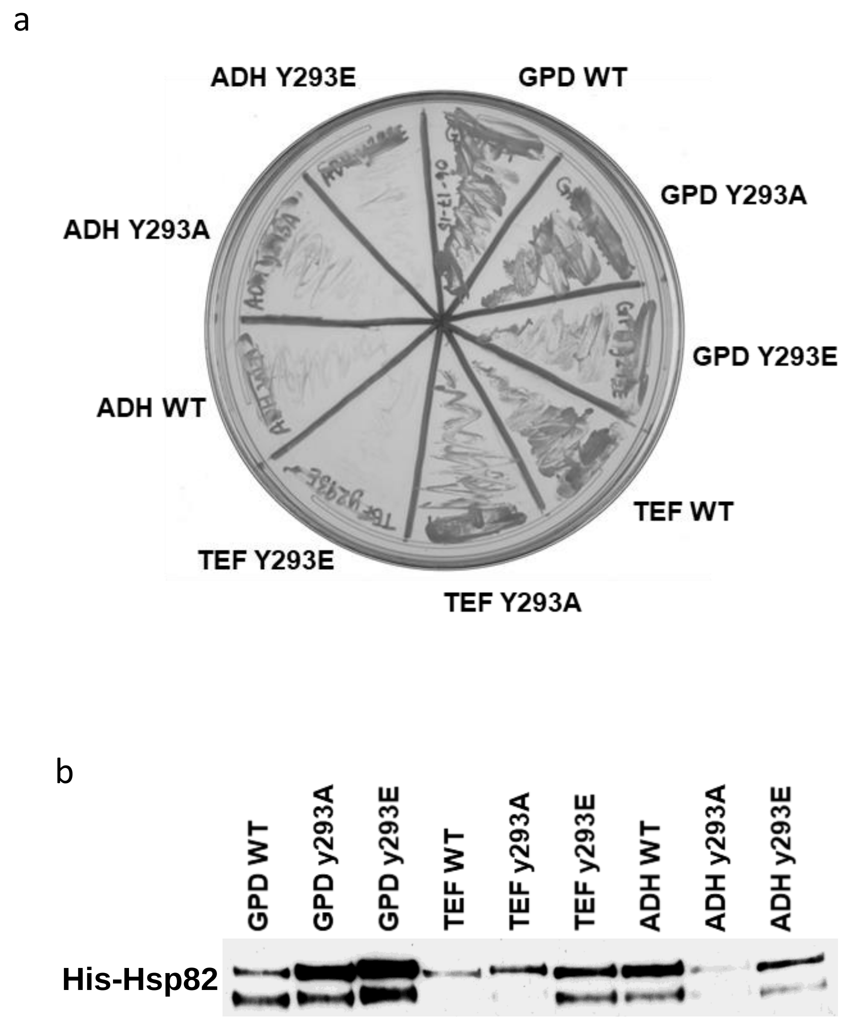

Supplementary Figure 6. Phosphomimetic substitution of the conserved Y313-equivalent amino acid in yeast Hsp82 (Y293) supports yeast growth when expressed from a GPD promoter. **a.** *hsc82hsp82* cells were transformed with the wild-type Hsp82 or mutant plasmids under the indicated promoter. Cells were then struck onto plates containing 5-FOA and grown for two days at 30° C. Hsp82 (wild-type, Hsp82-Y293A, Hsp82-Y293E) expressed from GPD promoter supported yeast growth; when expressed from TEF promoter, only wild-type and Hsp82-Y293A supported yeast growth. **b.** Cells grown on 5-FOA in (a) were grown overnight in rich media and lysed using glass beads. His-tagged Hsp82 proteins were isolated from lysate using nickel resin, run on a 4-20 % SDS gel and probed with Hsp82 antibody (Stress Marq Biosciences, Victoria, British Columbia, Canada; catalog No. SMC-135, clone Hyb-K41220A; dilution 1:1000). All samples contained an equal amount of protein (as determined by BCA assay). Under these conditions, the GPD promoter was markedly stronger than either TEF or ADH promoters. Based on the data in (a), Hsp82-Y293E, although less robust than wild-type or Hsp82-Y293A, supports yeast viability when strongly expressed. Source data for this figure are provided as a Source Data File.

Supplementary Table 1. A list of Hsp90 mutations used in this study. Included are Hsp90 domain location, major impact of each mutation on Hsp90, and relevant references.

Mutation	Domain of Hsp90	Impact on Hsp90	Reference
E47A	N	Able to bind but unable to hydrolyze ATP	(Grenert et al. 1999 [2])
D93A	N	Unable to bind ATP	(Grenert et al. 1999 [2])
K191R	N	Unable to be sumoylated, resulting in reduced Aha1 association	(Mollapour et al. 2014 [3])
H210A	N	Decreases association of Aha1 and p23, and the ATPase activity	This manuscript
Y313E	M	Increases Aha1 association	(Xu et al. 2012 [4])
W320A	M	Decreases Aha1-C interaction	(Meyer et al. 2003 [5])<br>This manuscript
R400A	M	Decreases ATP-induced association of the N- and M-domains of Hsp90	(Meyer et al. 2003 [5])<br>(Cunningham et al. 2012 [6])<br>This manuscript
V411E	M	Abrogates Aha1-N interaction	(Retzlaff et al. 2010 [7]),<br>This manuscript

Supplementary Table 2. Hsp90 H210A mutation decreases Hsp90 affinity for Aha1 by 2-fold while only modestly reducing the affinity for AMPPNP. Purified Hsp90-H210A protein was tested in vitro for AMPPNP and Aha1 affinities using isothermal titration calorimetry (ITC) [1]. The ITC buffer contained 20 mM Tris pH7.5, 5 mM NaCl, 1 mM EDTA, and the temperature was 30 °C. Experiments were performed a minimum of 3 times; Kd (µM) is shown as mean +/- S.D. Source data for this figure are provided as a Source Data File.

Protein	Ligand	N	Kd (µM)
Hsp90	AMPPNP	1.0 (fixed)	220±37
H210A	AMPPNP	1.0 (fixed)	170±9.8
Hsp90α	hAha1	0.36	7.1±1.3
H210A	hAha1	0.37	14.6±3.0

Supplementary Table 3. Phosphomimetic substitution of Y313 increases Hsp90 affinity for Aha1 protein. Human wild-type Hsp90, Hsp90-Y313E, and Aha1 (wild-type and individual domains) proteins were purified from E. coli. Affinity of purified Hsp90 proteins for Aha1 was determined *in vitro,* in the presence or absence of AMPPNP, using ITC. The ITC buffer contained 20 mM Tris, 100 mM NaCl, 0.5 mM EDTA, 5 mM $MgSO_4$ and 1 mM tris(2-carboxyethyl)phosphine, and the temperature was 36 ^{o}C. When indicated, AMPPNP was included in both the cell and the syringe at a concentration of 2 mM. The K_d for the interaction between wild-type Hsp90 and Aha1-C could not be determined because of the very low c-values obtained. Three technical repeats were performed for each titration; Kd values (µM) are shown as mean +/- S.D. Source data for this figure are provided as a Source Data File.

Proteins	– AMPPNP	+ AMPPNP
Hsp90-wt + Aha1	3.3 ± 0.5	1.9 ± 0.4
Hsp90-Y313E + Aha1	1.0 ± 0.3	1.1 ± 0.1
Hsp90-wt + Aha1-N	6.8 ± 0.6	4.9 ± 0.7
Hsp90-Y313E + Aha1-N	11.2 ± 1.2	8.9 ± 0.8
Hsp90-wt + Aha1-C	cannot be determined	15.6 ± 2.1
Hsp90-Y313E + Aha1-C	5.5 ± 2.0	2.3 ± 0.3

Supplementary Data 1. A list of all cross-linked peptide pairs assigned in this study (Excel file, attached separately). The list includes both target and decoy sequences which were used to estimate FDR. The 22 cross-linked peptide pairs quantified by PRM are highlighted in blue. For these 22 peptide pairs, the integrated peak areas, log2(AMPPNP/Apo) and corresponding 95% confidence intervals used for the charts in Figure 7, Supplementary Figure 3b and 3c, are included.

Supplementary References

1. Panaretou, B.; Siligardi, G.; Meyer, P.; Maloney, A.; Sullivan, J. K.; Singh, S.; Millson, S. H.; Clarke, P. A.; Naaby-Hansen, S.; Stein, R.; Cramer, R.; Mollapour, M.; Workman, P.; Piper, P. W.; Pearl, L. H.; Prodromou, C., Activation of the ATPase activity of hsp90 by the stress-regulated cochaperone aha1. *Mol Cell* **2002,** *10* (6), 1307-18.
2. Grenert, J. P.; Johnson, B. D.; Toft, D. O., The importance of ATP binding and hydrolysis by hsp90 in formation and function of protein heterocomplexes. *J Biol Chem* **1999,** *274* (25), 17525-33.
3. Mollapour, M.; Bourboulia, D.; Beebe, K.; Woodford, M. R.; Polier, S.; Hoang, A.; Chelluri, R.; Li, Y.; Guo, A.; Lee, M. J.; Fotooh-Abadi, E.; Khan, S.; Prince, T.; Miyajima, N.; Yoshida, S.; Tsutsumi, S.; Xu, W.; Panaretou, B.; Stetler-Stevenson, W. G.; Bratslavsky, G.; Trepel, J. B.; Prodromou, C.; Neckers, L., Asymmetric Hsp90 N domain SUMOylation recruits Aha1 and ATP-competitive inhibitors. *Mol Cell* **2014,** *53* (2), 317-29.
4. Xu, W.; Mollapour, M.; Prodromou, C.; Wang, S.; Scroggins, B. T.; Palchick, Z.; Beebe, K.; Siderius, M.; Lee, M. J.; Couvillon, A.; Trepel, J. B.; Miyata, Y.; Matts, R.; Neckers, L., Dynamic tyrosine phosphorylation modulates cycling of the HSP90-P50(CDC37)-AHA1 chaperone machine. *Mol Cell* **2012,** *47* (3), 434-43.
5. Meyer, P.; Prodromou, C.; Hu, B.; Vaughan, C.; Roe, S. M.; Panaretou, B.; Piper, P. W.; Pearl, L. H., Structural and functional analysis of the middle segment of hsp90: implications for ATP hydrolysis and client protein and cochaperone interactions. *Mol Cell* **2003,** *11* (3), 647-58.
6. Cunningham, C. N.; Southworth, D. R.; Krukenberg, K. A.; Agard, D. A., The conserved arginine 380 of Hsp90 is not a catalytic residue, but stabilizes the closed conformation required for ATP hydrolysis. *Protein Sci* **2012,** *21* (8), 1162-71.
7. Retzlaff, M.; Hagn, F.; Mitschke, L.; Hessling, M.; Gugel, F.; Kessler, H.; Richter, K.; Buchner, J., Asymmetric activation of the hsp90 dimer by its cochaperone aha1. *Mol Cell* **2010,** *37* (3), 344-54.
