# Supplementary material for: Hsp90 middle domain phosphorylation initiates a complex conformational program to recruit the ATPase-stimulating cochaperone Aha1
Source: Nat Commun. 2019 Jun 12;10:2574. doi: 10.1038/s41467-019-10463-y (PMC6561935; doi:10.1038/s41467-019-10463-y)
Supplement: Supplementary file 2 — Description of Additional Supplementary Files [file 41467_2019_10463_MOESM2_ESM.docx]

**Description of Additional Supplementary Files**

**File Name: Supplementary Data 1**

**Description:** A list of all cross-linked peptide pairs assigned in this study. The list includes both target and decoy sequences which were used to estimate FDR. The 22 cross-linked peptide pairs quantified by PRM are highlighted in blue. For these 22 peptide pairs, the integrated peak areas, log2(AMPPNP/Apo) and corresponding 95% confidence intervals used for the charts in Fig. 7, Supplementary Figure 3b and c are included.
