# Supplementary material for: Hsp90 middle domain phosphorylation initiates a complex conformational program to recruit the ATPase-stimulating cochaperone Aha1
Source: Nat Commun. 2019 Jun 12;10:2574. doi: 10.1038/s41467-019-10463-y (PMC6561935; doi:10.1038/s41467-019-10463-y)
Supplement: Supplementary file 4 — Reporting Summary [file 41467_2019_10463_MOESM4_ESM.pdf]

nature research

Corresponding author(s): Leonard Neckers

Last updated by author(s): Mar 7, 2019

Reporting Summary

Nature Research wishes to improve the reproducibility of the work that we publish. This form provides structure for consistency and transparency in reporting. For further information on Nature Research policies, see Authors & Referees and the Editorial Policy Checklist.

Statistics

For all statistical analyses, confirm that the following items are present in the figure legend, table legend, main text, or Methods section.

n/a	Confirmed	
☐	☒	The exact sample size (*n*) for each experimental group/condition, given as a discrete number and unit of measurement
☐	☒	A statement on whether measurements were taken from distinct samples or whether the same sample was measured repeatedly
☐	☒	The statistical test(s) used AND whether they are one- or two-sided<br>*Only common tests should be described solely by name; describe more complex techniques in the Methods section.*
☒	☐	A description of all covariates tested
☒	☐	A description of any assumptions or corrections, such as tests of normality and adjustment for multiple comparisons
☐	☒	A full description of the statistical parameters including central tendency (e.g. means) or other basic estimates (e.g. regression coefficient) AND variation (e.g. standard deviation) or associated estimates of uncertainty (e.g. confidence intervals)
☒	☐	For null hypothesis testing, the test statistic (e.g. *F*, *t*, *r*) with confidence intervals, effect sizes, degrees of freedom and *P* value noted<br>*Give P values as exact values whenever suitable.*
☒	☐	For Bayesian analysis, information on the choice of priors and Markov chain Monte Carlo settings
☒	☐	For hierarchical and complex designs, identification of the appropriate level for tests and full reporting of outcomes
☒	☐	Estimates of effect sizes (e.g. Cohen's *d*, Pearson's *r*), indicating how they were calculated

Our web collection on statistics for biologists contains articles on many of the points above.

Software and code

Policy information about availability of computer code

Data collection	Western blotting--Adobe Photoshop; LC-MS--Q-Exactive Plus mass spectrometer
Data analysis	LC-MS--analysis via Comet v. 20198.01.2 and Skyline

For manuscripts utilizing custom algorithms or software that are central to the research but not yet described in published literature, software must be made available to editors/reviewers. We strongly encourage code deposition in a community repository (e.g. GitHub). See the Nature Research guidelines for submitting code & software for further information.

Data

Policy information about availability of data

All manuscripts must include a data availability statement. This statement should provide the following information, where applicable:

- Accession codes, unique identifiers, or web links for publicly available datasets
- A list of figures that have associated raw data
- A description of any restrictions on data availability

Figures and tables with associated raw data are identified in the respective legends and the Data Availability section. All relevant data are available from the authors upon reasonable request.

Field-specific reporting

Please select the one below that is the best fit for your research. If you are not sure, read the appropriate sections before making your selection.

☒ Life sciences ☐ Behavioural & social sciences ☐ Ecological, evolutionary & environmental sciences

October 2018

Life sciences study design

All studies must disclose on these points even when the disclosure is negative.

Sample size	All experiments were repeated at least 3 times and means +/- standard deviations were reported where appropriate.
Data exclusions	No data were excluded from analysis.
Replication	There were at least three biological replicates of each experiment.
Randomization	Randomization is not relevant to this study, as different mutations were compared to wild type, making randomization impossible.
Blinding	Blinding is not relevant to this study, for the reason listed above.

Reporting for specific materials, systems and methods

We require information from authors about some types of materials, experimental systems and methods used in many studies. Here, indicate whether each material, system or method listed is relevant to your study. If you are not sure if a list item applies to your research, read the appropriate section before selecting a response.

Materials & experimental systems

n/a	Involved in the study
☐	☒ Antibodies
☐	☒ Eukaryotic cell lines
☒	☐ Palaeontology
☒	☐ Animals and other organisms
☒	☐ Human research participants
☒	☐ Clinical data

Methods

n/a	Involved in the study
☒	☐ ChIP-seq
☒	☐ Flow cytometry
☒	☐ MRI-based neuroimaging

Antibodies

Antibodies used	mouse anti-FLAG, clone M2, cat. # F3165-1MG, Sigma; mouse anti-HA, clone HA-7, cat. # H3663, Sigma; rat monoclonal anti-HA, clone 3F10, cat. # 11867423001, Sigma; rabbit anti-Hsp90, clone C45G5, cat. # 4877S, Cell Signaling; rabbit anti-Hop, clone D10E2, cat. # 5670S, Cell Signaling; rabbit DYKDDDDK Tag Polyclonal Antibody, cat. # PA1-984B, ThermoFisher Scientific; rat anti-Hsp90, clone 16F1, cat. # ADI-SPA-835, Enzo Life Sciences; rabbit anti-Hsp90alpha, cat. # ADI-SPS-771, Enzo Life Sciences; mouse anti-Hsp90, clone AC88, cat. # ADI-SPA-830, Enzo Life Sciences, mouse anti-Hsp90, clone H9010, a gift; rabbit anti-p23, cat. # ADI-SPA-670, Enzo Life Sciences; rabbit anti-Aha1, cat. # 600-401-974, Rockland Immunochemicals; site-specific rabbit anti-Hsp90alpha phospho-Y313; Cell Signalling (collaboration). All primary antibodies were used at 1:1000 dilution. HRP-conjugated secondary antibodies were used at a 1:5000 dilution.
Validation	All antibodies are validated according to manufacturer information.

Eukaryotic cell lines

Policy information about cell lines

Cell line source(s)	HEK-293 and HEK-293A
Authentication	HEK-293 purchased from ATTC and HEK-293A were purchased from Invitrogen and authentication was performed by the company.
Mycoplasma contamination	The companies confirmed both cell lines were free of mycoplasma contamination.
Commonly misidentified lines (See ICLAC register)	not relevant to this study
